# 4-Phenylbutyrate restores localization and membrane repair to human dysferlin mutations

**DOI:** 10.1016/j.isci.2021.103667

**Published:** 2021-12-20

**Authors:** Kana Tominaga, Naoomi Tominaga, Eric O. Williams, Laura Rufibach, Verena Schöwel, Simone Spuler, Mohan Viswanathan, Leonard P. Guarente

**Affiliations:** 1 Paul F. Glenn Center for Biology of Aging, Department of Biology, Koch Institute, Massachusetts Institute of Technology, Cambridge, MA 02139, USA; 2 Department of Pathology and Oncology, Juntendo University School of Medicine, 2-1-1 Hongo, Bunkyoku, Tokyo 113-8421, Japan; 3 Fitchburg State University, School of Heath and Natural Sciences, Antonucci Science Complex 235, Fitchburg, MA 01420, USA; 4 Jain Foundation, 9706 4th Avenue NE, Suite 101, Seattle, WA 98115, USA; 5 Muscle Research Unit, Experimental and Clinical Research Center (ECRC), a joint cooperation of Charité Universitätsmedizin Berlin and Max-Delbrück Center for Molecular Medicine, Berlin, Germany

**Keywords:** Musculoskeletal medicine, Drugs

## Abstract

Dysferlinopathies are muscular dystrophies caused by recessive loss-of-function mutations in dysferlin (*DYSF*), a membrane protein involved in skeletal muscle membrane repair. We describe a cell-based assay in which human DYSF proteins bearing missense mutations are quantitatively assayed for membrane localization by flow cytometry and identified 64 localization-defective DYSF mutations. Using this platform, we show that the clinically approved drug 4-phenylbutryric acid (4-PBA) partially restores membrane localization to 25 mutations, as well as membrane repair to cultured myotubes expressing 2 different mutations. Two-day oral administration of 4-PBA to mice homozygous for one of these mutations restored myofiber membrane repair. 4-PBA may hold therapeutic potential for treating a subset of humans with muscular dystrophy due to dysferlin deficiency.

## Highlights

• Most dysferlinopathy patient missense mutants do not localize to the plasma membrane
• 4-PBA and corr-2b restore membrane localization to patient DYSF missense mutations
• 4-PBA restores membrane repair to muscle fibers from MMex38 dysferlin mutant mice
• Two-day oral treatment with 4-PBA restores mutant DYSF function in mouse muscle

## Graphical abstract

**Figure:**
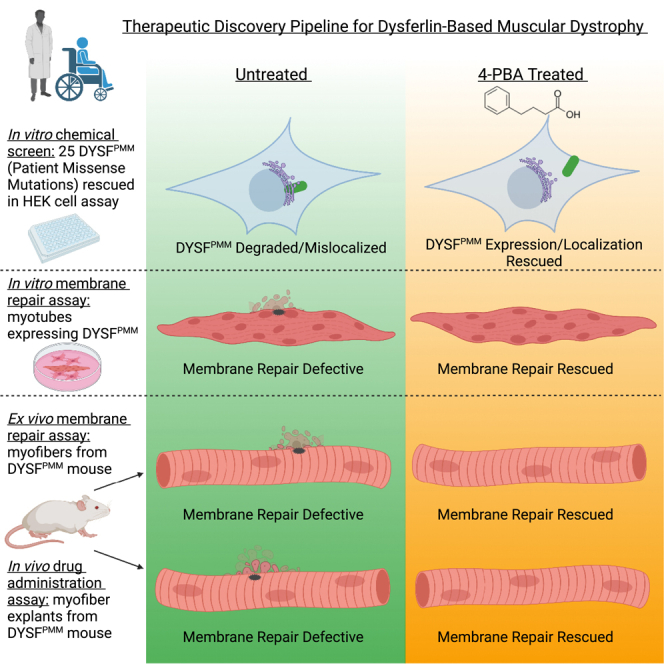

## Introduction

Dysferlinopathy is an adult-onset, progressive, rare form of muscular dystrophy caused by recessive loss-of-function mutations in the gene encoding dysferlin, *DYSF,* and includes the clinical diagnoses of limb-girdle muscular dystrophy 2B/R2, Miyoshi myopathy type 1, and distal anterior compartment myopathy (Illa et al., 2001; Liu et al., 1998). A diagnosis of dysferlinopathy is made when a patient is shown to have >80% reduction in DYSF protein by western blot (Cacciottolo et al., 2011) and is confirmed by sequencing of the *DYSF* gene to identify the causative mutation(s). Patients typically lose ambulation by age 40–45 years (Fanin and Angelini, 2016; Harris et al., 2016), and there are no current treatments.

Dysferlin (DYSF) is a member of the Ferlin protein family found throughout metazoans (Lek et al., 2010). A tail-anchored type-2 integral plasma membrane (PM) protein, DYSF has a very short extracellular domain and a large intracellular domain containing 7 calcium-binding C2-domains (Abdullah et al., 2014), as well as FerA and DysF domains that together mediate intracellular membrane fusion events (Bansal and Campbell, 2004; Lek et al., 2012), calcium homeostasis (Kerr et al., 2014), and lipid metabolism (Haynes et al., 2019). DYSF is expressed in skeletal muscle where it mediates Ca^2+^-dependent vesicle fusion and repair of sarcolemma following the creation of membrane breaches by tensile forces during muscle contractions (Bansal et al., 2003). Other reported roles for dysferlin include calcium regulation at the t-tubule triad junction (Kerr et al., 2014), vesicular trafficking (Bansal et al., 2003; Lek et al., 2012), and monocyte adhesion (de Morree et al., 2013).

We developed a therapeutic pipeline to identify small molecules capable of restoring function to loss-of-function *DYSF* patient missense mutations (DYSF^PMMs^), which represent 30%–40% of dysferlinopathy mutations (Cacciottolo et al., 2011; Jin et al., 2016; Schoewel et al., 2012). Since dysferlinopathy is a recessive disorder, restoration of missense-allele function is likely therapeutic for patients with at least one such allele. One of the well-characterized DYSF^PMMs^, DYSF^L1341P^, shows protein misfolding, aggregation in the endoplasmic reticulum (ER), and degradation by the proteasome (Schoewel et al., 2012). We surmised that this and other DYSF^PMMs^ may be a class of unstable endomembrane trafficking defective mutants that could be rescued by chemical chaperones or correctors.

## Results

### Development of a quantitative DYSF membrane localization assay

**Figure 1:**
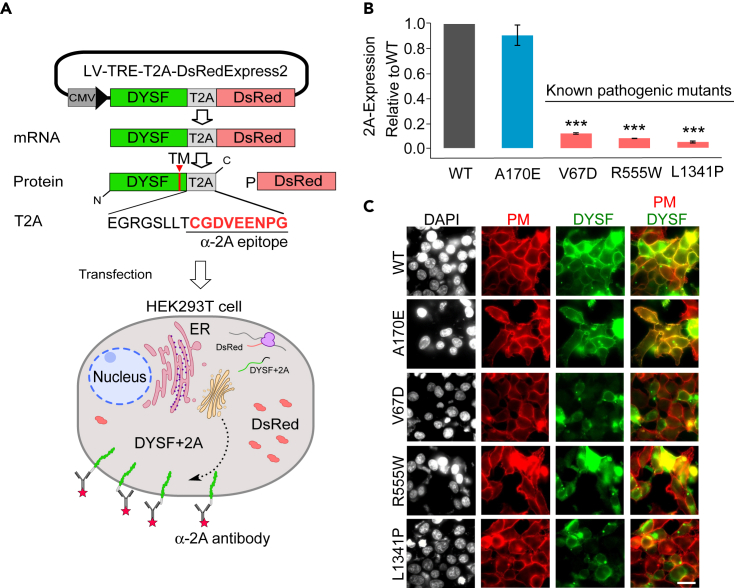
Determination of DYSF^PMMs^ PM localization: The 2A-assay (A) Schematic outline of the 2A-assay. Human DYSF isoform 8 cDNA was inserted into a bicistronic lentiviral vector expression vector regulated by a minimal cytomegalovirus (CMV) promoter and tetracycline response element (TRE). Transfection of LV-TRE-DYSF-T2A-DsRed into HEK293T cells results in equimolar expression of DYSF-2A, a fusion protein with a C-terminal 2A-peptide, and DsRed protein, which are separated by cleavage at the T2A peptide sequence during translation. A mouse α-2A peptide antibody recognizes the extracellular region of DYSF-T2A protein in live cells; subsequent binding of Alexa 647 α-mouse IgG secondary antibody enables quantitation of PM-localized DYSF intensity on the surface of live cells that express cytoplasmic DsRed by flow cytometry. The amount of DsRed translationally expressed is equimolar to the amount of DYSF-T2A. Quantification of these 2 signals allows us to calculate a “2-A assay value,” which represents the amount of PM-localized Dysferlin relative to DsRed for any HEK cell population expressing a mutant DYSF relative to the 2-A assay value in a similar population of cells expressing wild-type DYSF. (B) Validation of the HEK cell-based 2A-assay was performed using vector constructs expressing DYSF^WT^ and 1 of the following 3 known pathogenic DYSF^PMMs^: DYSF^V67D^, DYSF^R555W^ and DYSF^L1341P^, and DYSF^A170E^, a commonly occurring DYSF SNP that is predicted to be non-pathogenic. HEK cells were transiently transfected, cultured, and processed for flow cytometry as described in materials and methods. PM localized expression of DYSF^PMMs^ is determined relative to DYSF^WT^. Data are represented as mean (n = 3) ± S.D., ∗∗∗p < 0.001 versus DYSF^WT^, by Student’s t test. (C) Immunofluorescence images localizing DYSF^PMMs^ expressed in HEK cells. Forty-eight hours post transfection HEK cells expressing the listed DYSF^PMMs^ were FACS sorted for DsRed, cultured, fixed, and stained with the Hamlet α-DYSF 1˚Ab (green) and the α-Na/K ATPase-Ab (red) to identify the plasma membrane (PM). Scale bar: 25 μm.

To characterize a large number of DYSF^PMMs^ for membrane trafficking defects, we created a cell-based flow cytometry assay that quantifies the amount of any given PM-localized DYSF^PMM^ relative to DYSF^WT^. Briefly, LV-TRE-DYSF-T2A-DsRed, a bicistronic expression vector with an intervening T2A translational cleavage sequence (Kabadi et al., 2015), was constructed with human DYSF^WT^ upstream of the fluorescent reporter gene *DsRed* (Figure 1A) allowing equimolar expression of both genes from a single mRNA. Transfection of this construct into human embryonic kidney (HEK)293T cells results in expression of DYSF^WT^-T2A, a fusion protein with a small C-terminal 2A-epitope exposed on the cell surface (Figure 1A) allowing for the unique detection (Figures 3B and S1A) and quantitation (Figure S1B) of PM-resident DYSF (Figure 3B) in live cells by flow cytometry using an anti(α)-2A antibody (Ab); we term this the *2A-assay*. In addition to DYSF^WT^, 113 DYSF^PMMs^ were selected with assistance from the Jain Foundation Dysferlin Registry and engineered into the bicistronic vector system for 2A-assay analysis (Table S1).

Initially, 3 known pathogenic DYSF^PMMs^, DYSF^V67D^ (Illarioshkin et al., 2000), DYSF^R555W^ (Nguyen et al., 2005), and DYSF^L1341P^ (Fujita et al., 2007; Malcher et al., 2018; Schoewel et al., 2012), that show little to no DYSF protein in patient muscle biopsies and the frequently occurring (1% allele frequency, dbSNP: rs34999029) non-pathogenic DYSF SNP, DYSF^A170E^ (Nguyen et al., 2005), were examined by 2A-assay. The ratio of PM-localized mutant DYSF^PMM^ relative to DYSF^WT^ among transfected DsRed-positive cells was quantified by flow cytometry; although no significant difference was observed between DYSF^WT^ and DYSF^A170E^, less than 14% of DYSF^V67D^, DYSF^R555W^, or DYSF^L1341P^ was found at the cell surface (Figure 1B). We performed fluorescent immunocytochemical (ICC) analysis on similarly transfected cells selected by fluorescence-activated cell sorting (FACS) for DsRed. Cells were fixed and treated with DAPI, α-DYSF Ab, α-Na/K ATPase Ab, and an appropriate fluorescently conjugated secondary Ab in order to localize nuclei, DYSF, and HEK cell PM, respectively. As expected, DYSF^A170E^ was localized to the plasma membrane similar to DYSF^WT^, whereas the known pathogenic DYSF^PMMs^ showed substantially reduced expression at the PM (Figure 1C), supporting our 2A-assay results. In cells where discrete intracellular DYSF localization was detected, it was often perinuclear and showed colocalization with an ER marker (Figure S2). We surmise this represents aggregation of mutant proteins, as previously reported for DYSF^L1341P^ (Fujita et al., 2007; Schoewel et al., 2012) and other DYSF^PMMs^. A small percentage of cells show indiscriminate ICC staining or non-staining, which we think corresponds to over- or under-cell permeabilization, which is not carried out in the live-cell 2A-assay.

### Identification of PMMs that prevent DYSF PM-localization

**Figure 2:**
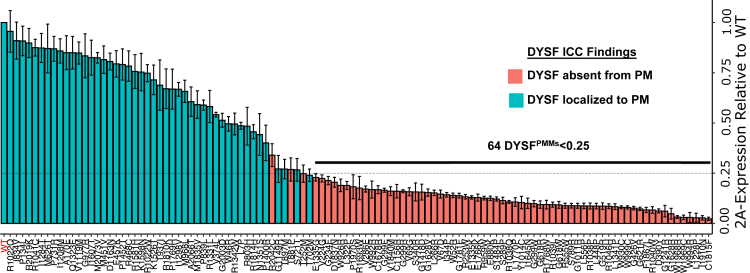
Determination of PM-localized expression of 113 DYSF^PMMs^ by 2A assay and ICC HEK cells were transiently transfected with LV-TRE-DYSF-T2A-DsRed vectors bearing DYSF^WT^ or 1 of 113 various DYSF^PMMs^. The amount of PM-localized DYSF was determined by 2A-assay; expression of PMMs is reported relative to DYSF^WT^ (n = 6. Data are means ± S.D.). DsRed-positive cells were sorted and cultured on coverslips and subject to ICC to visually determine DYSF localization; red bars indicate no observable PM localization, whereas blue bars indicate that PM localization was observed for a given DYSF^PMM^.

One hundred thirteen DYSF^PMMs^ from a subset of 327 patients with dystrophic phenotypes consistent with dysferlinopathy from the Jain Foundation Dysferlin Registry were cloned into our bicistronic expression vector system (Figure S3). The results of 2A-assays and ICC-based localizations for the 113 DYSF^PMMs^ are tabulated in Table S1 and displayed graphically in Figure 2; examples of ICC images for DYSF^PMMs^ with various 2A-assay expression levels are shown in Figure S4. There is a high degree of correspondence between both assays in detecting DYSF^PMMs^ at the PM of HEK cells, with a transition zone of ICC detection of PM-localized DYSF at 25% of wild-type DYSF levels by 2A-assay. Three PMMs with discrepancies between these assays (T252M, T881P, and R2042C) were found around this 25% level and are likely the result of the greater accuracy of machine detection over visual-based scoring methods at this level of fluorescent sensitivity. When plotted as a positional lolliplot (Figure S5), we find that mutations that disrupt PM localization are distributed across DYSF, with no region noticeably incapable of affecting localization when mutated.

We identified 64 of the 113 DYSF^PMMs^ below the 25% 2A-assay threshold (Figure 2) and predict them to be pathogenic, most likely due to DYSF protein misfolding leading to protein mislocalization, aggregation, and/or degradation. The identification of a mechanism of pathogenesis for DYSF^PMMs^ is likely to aid clinicians in the diagnosis of individuals with dysferlinopathy through the (re)classification of known DYSF missense variants as pathogenic.

Of 36 DYSF^PMMs^ that are found to be homozygous in 90 individuals from the Dysferlin Registry (Table S2), 26 (72%) have 2A-expression levels less than 25% of DYSF^WT^ and are absent from the PM by ICC, indicating that this class holds predicative value for determining PMM pathogenicity. The remaining 10 PMMs have 2A-assay values above the 25% threshold but less than DYSF^WT^ (Table S2), and 9 are visually localized to the PM by ICC. R2042C is not, suggesting it is likely pathogenic (Figures 2 and S5). Of the 9 mutants showing PM localization by ICC, 2 have been shown or predicted to cause exon skipping (R2019K and R1810K) and produce nonfunctional proteins and 3 DYSF mutants, I1607T, R1331L, and K1526T, are likely being benign; as I1607T and R1331L are found in individuals with other homozygous PMMs with 2A-values <0.25, and K1526T is cis with another pathogenic DYSF variant.

There are an additional 38 DYSF^PMMs^ with 2A-assay values >25% DYSF^WT^ found in individuals who are compound heterozygote carrying at least 1 DYSF^PMM^ (Table S3). Thirty of these have evidence of non-pathogenicity (Table S3): 3 have been found in patients confirmed to have another form of muscular dystrophy, 8 were found in patients with normal or heterozygous carrier levels of DYSF protein and therefore likely do not have dysferlinopathy, 4 are either predicted or proven splicing defects, 11 are found in patients with 2 or more other known pathogenic mutations, and 4 are confirmed as benign by ClinVar.

### Development and validation of a therapeutic screening platform

We developed a 96-well 2A-assay-based chemical screening platform to identify compounds capable of restoring PM localization to the 64 DYSF^PMMs^ with 2A-assay values <25% of DYSF^WT^. We first tested 10 compounds previously reported to rescue misfolding of other disease-relevant proteins in the ER, such as the cystic fibrosis transmembrane conductance regulator (CFTR) (Pedemonte et al., 2005) and α-sarcoglycan (Carotti et al., 2018, 2020). We also tested the drug 4-phenylbutyric acid (4-PBA), used for the treatment of urea cycle disorder diseases, which has also been proposed to act as a chemical chaperone for diseases involving misfolded missense mutants (Kolb et al., 2015), such as the Ryanodine Receptor (RYR) (Lee et al., 2017) and the CFTR Δ508 mutant (Rubenstein et al., 1997; Rubenstein and Zeitlin, 1998), and an ER-stress reducer improving outcomes in amyotrophic lateral sclerosis (ALS) (Paganoni et al., 2020) and myopathies (Zito, 2019). This compound has also been reported to increase expression of the DYSF^R959W^ mutant and rescue its localization to t-tubules (Bersch, 2017).

**Figure 3:**
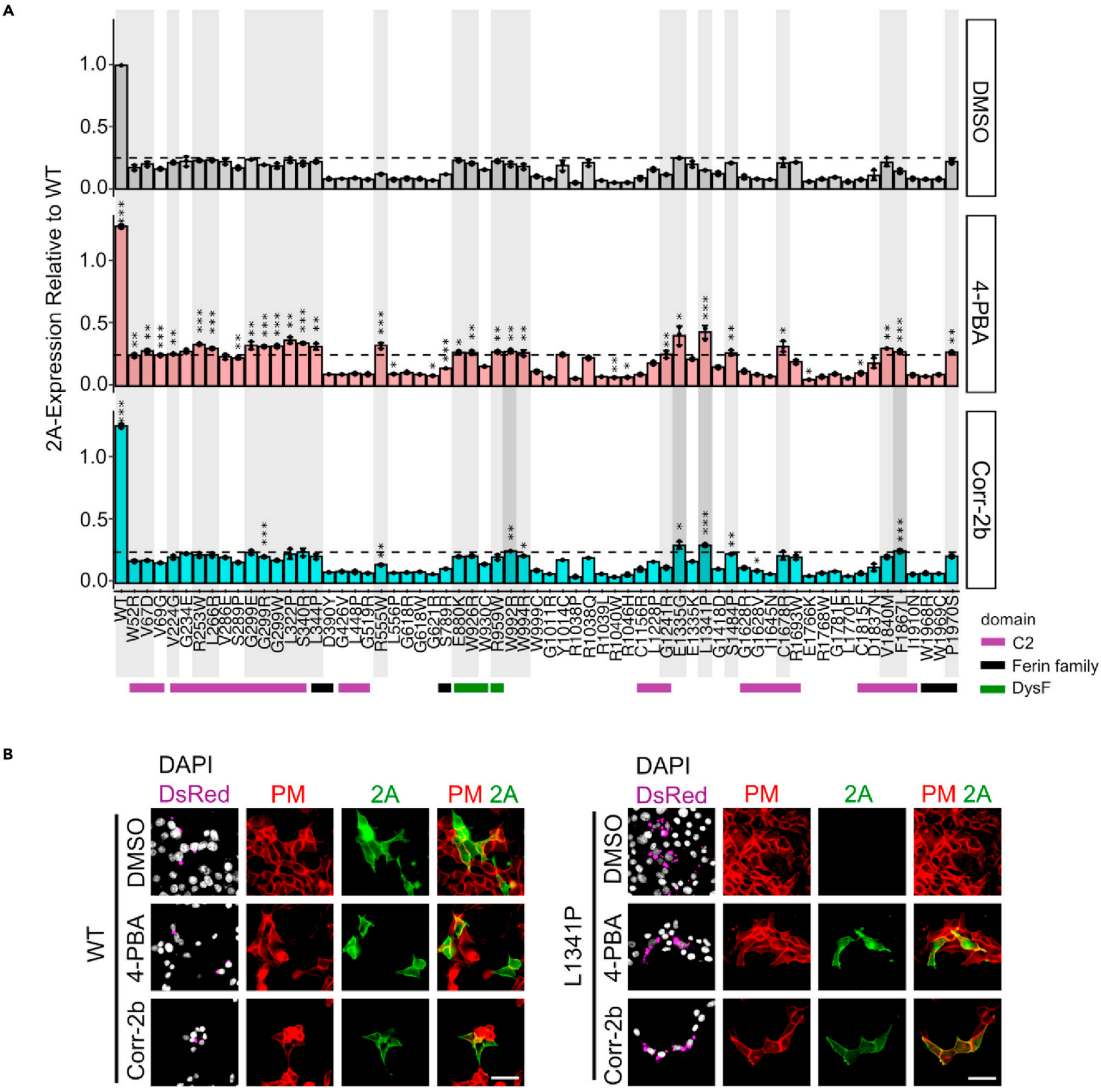
Restoration of DYSF^L1341P^ PM localization by 4-PBA and corr-2b (A) Results of 2A-assays (n = 3, data are means ± S.D.) on 64 DYSF^PMMs^ that have less than 25% of DYSF^WT^ PM localization (Figure 2) following treatment with DMSO (0.1%), 4-PBA (1 mmol/L), or corr-2b (25 μmol/L) for 24 h. Twenty-one mutants (highlighted in light gray) significantly respond to 4-PBA only and 4 mutants (highlighted in dark gray) responded to both 4-PBA and corr-2b, boosting DYSF^PMMs^ PM localization above the 25% threshold (dashed line). ∗p < 0.05, ∗∗p < 0.01, ∗∗∗p < 0.001 by Student t-test for all figures. (B) ICC DYSF localization in transfected (unsorted) HEK cells expressing DsRed and either DYSF^WT^ or DYSF^L1341P^ treated with DMSO (0.1%), 4-PBA (1 mM), or corr-2b (25 μM) for 24 h. Live cells were stained with α-2A-Ab (green) to identify PM-localized DYSF protein. DAPI staining and α-Na/K ATPase-Ab (red) hybridization was used to identify the nuclei and PM in all cells, respectively. Scale bar: 50 μm.

HEK cells transiently expressing DYSF^WT^, or the 64 DYSF^PMMs^ mutants previously found to be below 25% of DYSF^WT^ in 2A-assays, were DsRed (+) sorted and treated with compounds or DMSO vehicle control for 24 h prior to the 2A-assay and scored for elevated expression of PM-localized DYSF^PMMs^ above 25% (Figure 3A). 4-PBA boosted expression above the 25% threshold in 25/64 DYSF^PMMs^ (Figure 3A). Although 4-PBA did not restore PM localization to wild-type levels, the restoration was statistically significant and in some cases resulted in full membrane repair activity, as we show below. Among CFTR corrector compounds, we found corr-2b rescued DYSF^W992R^, DYSF^E1335G^, DYSF^L1341P^, and DYSF^F1867L^ (Figure 3A). These 4 DYSF^PMMs^ were also rescued and to a greater degree by 4-PBA. The restoration of DYSF^L1341P^ PM localization by 4-PBA and corr-2b was confirmed by ICC (Figure 3B); cells expressing DYSF^WT^ treated with vehicle showed PM localization by ICC but DYSF^L1341P^ expressing cells did not; however, 4-PBA and corr-2b treatment restored PM localization of DYSF^L1341P^ (Figure 3B). We found that 4-PBA and corr-2b negatively impacted HEK cell proliferation at doses above 1 mM and 25 μM, respectively (Figure S6A), while showing efficacy in restoring PM localization to DYSF^L1341P^ in 2A-assays at or below these concentrations (Figure S6B). The effects of 4-PBA and corr-2b were not additive (Figure S6C), suggesting they may work in the same pathway. Mutations in C2 domains appeared to be rescued by 4-PBA and/or corr-2b at a much higher frequency ([46%] 19/41 mutations) than mutations in other regions ([26%] 6/23 mutations) (Figure S7).

Of interest, PM localization of DYSF^WT^ was 20% greater after treating cells with 4-PBA or corr-2b (Figures 3A and S6B). Western analysis revealed no obvious change in protein levels of DYSF^WT^ or DYSF^L1341P^ by either compound (Figure S6D). In summary, 4-PBA and corr-2b may work to aid proper DYSF folding, stability, transport through the endomembrane system to the PM, and possibly endocytic recycling of protein from the PM (Evesson et al., 2010).

### Functional assay: membrane repair in muscle myotubes expressing DYSF^PMMs^

GREG cells are a naturally immortalized DYSF-deficient mouse myoblast cell line derived from A/J mice (Humphrey et al., 2012). In addition, GREG cells transfect well and are facile for differentiation into myotubes. In order to determine if the hit compounds found in the 2A-assay chemical screen are efficacious in restoring DYSF^PMM^ function we developed a functional assay using *in vitro* differentiated GREG myotubes transfected with DYSF expression vectors. DsRed-positive GREG cells were isolated by FACS following transfection with either DYSF^WT^ or DYSF^L1341P^ expression vectors and plated in media to promote myoblast fusion. The resulting myotubes were assayed for DYSF-mediated membrane repair capacity following laser damage in the presence of the membrane impermeant fluorescent dye FM1-43 and calcium (Figure S8A). The repair deficiency of non-transfected DYSF-deficient GREG myotubes was evidenced by the rapid and continuous FM1-43 dye influx following membrane laser wounding (Figure S8B, Video S1). In contrast, rapid membrane resealing was observed in GREG myotubes expressing DYSF^WT^ (Figure S8, Video S2). (In earlier assay development in C2C12 cells, no repair was seen in the absence of calcium [not shown].) Membrane wounding of GREG myotubes expressing DYSF^L1341P^ resulted in rapid and sustained dye influx indicating significant repair deficiency (Figure S8, Video S3). GREG myotubes expressing DYSF^L1341P^ did display some residual repair capacity compared with non-transfected myoblasts (Figure S8B); high-level expression of DYSF^L1341P^ in GREG cells could result in residual membrane localization of this mutant, which is known to be repair proficient when at the membrane (Schoewel et al., 2012).

Video S1. GREG-untransfected.avi, related to Figure S8A

Video S2. GREG-WT.avi, related to Figure S8A

Video S3. GREG-L1341P.avi, related to Figure S8A

### Efficacy of compounds on muscle membrane repair in mice

**Figure 4:**
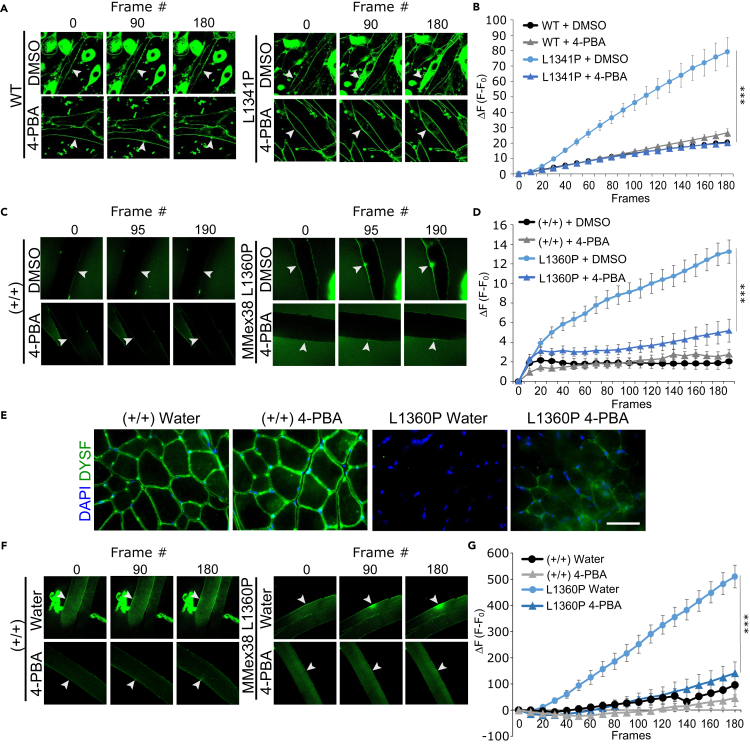
Treatment with 4-PBA restores membrane repair in GREG (DYSF^L1341P^) myotubes and MMex38 (DYSF^L1360P^) mouse myofibers (A–D) Selected image frames from beginning, middle, and end of membrane repair assays in the presence of FM1-43 dye following laser irradiation of myotube (A) or myofiber (C and F) membranes; white arrowheads show sites of membrane wounding by laser. Quantification of the change in fluorescent intensity (ΔF) caused by intracellular FM1-43 dye infiltration after membrane injury (B, D, and G); n-values are the total number of fibers tested in 2 independent experiments. (A and B) DYSF^WT^ or DYSF^L1341P^ transfected GREG myotubes treated with listed compounds; DYSF^WT^ (DMSO) n = 15, DYSF^WT^ (4-PBA) n = 19, DYSF^L1341P^ (DMSO) n = 16, DYSF^L1341P^ (4-PBA) n = 26. (C and D) C57BL6/NJ (+/+) or MMex38 (L3160P) mouse myofibers from explanted EDL muscles treated with DMSO or 4-PBA (1 mM) *in vitro* for 24 h; fiber number; +/+ (DMSO) n = 6, +/+ (4-PBA) n = 16, L1360P (DMSO) n = 15, L1360P (4-PBA) n = 18. (E–G) (E) Immunofluorescent staining of fresh frozen histological cross sections of EDL muscle isolated from 3-month-old male +/+ and MMex38 mice treated with vehicle or 4-PBA (2 mg/mL) in drinking water for 48 h. DAPI staining was performed for nuclear localization, and DYSF staining was done using Romeo α-DYSF-1 Ab and Alexa 647 α-mouse IgG Ab fluorescent secondary Ab. Images were all taken at the same exposure time and magnification; scale bar, 50 μm. Representative assay images (F) and membrane repair kinetics (G) of EDL muscle myofibers isolated from +/+ and MMex38 (L1360P) mice treated with vehicle or 4-PBA in drinking water for 48 h. Number and genotype of mice treated; +/+ (water) n = 2, +/+ (4-PBA) n = 2, L1360P (water) n = 3, L1360P (4-PBA) n = 3; Number and genotype of fibers used for membrane repair assay; +/+ (water) n = 9, +/+ (4-PBA) n = 5, L1360P (water) n = 17, L1360P (4-PBA) n = 22. All plotted data are means ± S.D; p values calculated by Student’s t test (∗∗∗p < 0.001).

We next evaluated the efficacy of the hit compounds found from our primary screening platform in functional assays of membrane repair in muscle. First, myotubes were generated from transfected DsRed^+^ GREG myoblasts expressing DYSF^WT^, DYSF^R555W^, and DYSF^L1341P^ and assayed for membrane repair following laser wounding. The 2 mutants chosen both localized to the PM in HEK cells following treatment with 4-PBA, and DYSF^L1341P^ additionally responded to corr-2b (Figure 3). As expected, when expressed in GREG myotubes, both mutants were membrane repair defective similar to vehicle treatment (Figures 4A, 4B, and S8). In contrast, 24-h treatment with 1 mM 4-PBA rescued membrane resealing following injury in GREG myotubes expressing DYSF^R555W^ (Figure S9) or DYSF^L1341P^ (Figures 4A and 4B, Videos S4, S5, S6, and S7). At the dose tested, corr-2b appeared to exacerbate laser wounding damage of myotubes expressing DYSF^WT^ (Figure S10), precluding assessment of any rescue of DYSF^L1341P^. We thus focused on 4-PBA in determining whether a compound could restore membrane resealing in skeletal muscle *in vivo*.

Video S4. GREG-WT-DMSO.avi, related to Figure 4A

Video S5. GREG-WT-4PBA.avi, related to Figure 4A

Video S6. GREG-L1341P-DMSO.avi, related to Figure 4A

Video S7. GREG-L1341P-4PBA.avi, related to Figure 4A

MMex38 (C57BL/6N;129P2-Dysf^tm1.1Mdcb^) mice are homozygous for mDYSF^L1360P^, which is analogous to the PMM DYSF^L1341P^. Histologically, MMex38 mice have no PM DYSF expression in mouse skeletal muscle and display age-progressive dystrophic histological phenotypes consistent to that seen in patients with dysferlinopathy (Figure S11, Malcher et al., 2018). Extensor digitorum longus (EDL) muscle from MMex38 and C57BL/6NJ (+/+) control animals were explanted and treated in culture with vehicle or 1 mM 4-PBA for 24 h. DYSF^L1360P^ was completely absent in histological cross-sections of vehicle-treated EDL muscle; however, DYSF^L1360P^ expression and localization to muscle sarcolemma was partially restored in EDL muscle explants after 24-h treatment with 1 mM 4-PBA (Figure S12). Although myofibers from untreated MMex38 EDL explants were membrane repair deficient following laser injury, 24-h treatment with 4-PBA fully restored membrane resealing function similar to EDL myofibers from either treated or untreated age-matched wild-type animals (Figures 4C and 4D, Videos S8, S9, S10, and S11).

Video S8. (+/+) -EDL-DMSO.avi, related to Figure 4C

Video S9. (+/+) -EDL-4PBA.avi, related to Figure 4C

Video S10. L1360P-EDL-DMSO.avi, related to Figure 4C

Video S11. L1360P-EDL-4PBA.avi, related to Figure 4C

We next administered 4-PBA (2 mg/mL) for 48 h in the drinking water of MMex38 and control animals and assayed DYSF localization and membrane repair in EDL muscle explants. Histological cross sections of EDL muscle from wild-type animals (+/+) showed clear sarcolemma localization of DYSF^WT^, which was enhanced by 24-h 4-PBA treatment (Figure 4E), similar to HEK cells expressing DYSF^WT^ treated with 4-PBA above. Histological cross sections of EDL tissue from untreated MMex38 mutant mice were devoid of DYSF staining, but 2 days of 4-PBA administration partially restored DYSF^L1360P^ myofiber sarcolemma localization as seen in DYSF staining of EDL cross sections (Figure 4E).

As expected, muscle fiber repair activity was robust in both 4-PBA-treated and untreated wild-type mice (Figures 4F and 4G, Videos S12 and S13). As above, EDL muscle fibers from untreated MMex38 mutant mice were devoid of EDL myofiber membrane repair activity (Figures 4F and 4G, Video S14). However, 2 days of 4-PBA administration fully restored membrane repair activity to mutant myofibers (Figures 4F and 4G, Video S15), resulting in membrane repair kinetics similar to that of wild-type animals. We therefore conclude that oral administration of 4-PBA is robustly effective in rescuing the defects of DYSF^L1360P^ (human DYSFL^L1341P^) and may also restore activity to other DYSF^PMMs^.

Video S12. (+/+) -water.avi, related to Figure 4F

Video S13. (+/+) -4PBA.avi, related to Figure 4F

Video S14. L1360P-water.avi, related to Figure 4F

Video S15. L1360P-4PBA.avi, related to Figure 4F

## Discussion

We describe a cell-based platform for identifying defective dysferlin missense mutations based on their inability to localize and accumulate in the plasma membrane. We identified 64 of 113 DYSF^PMMs^ that fall below 25% of DYSF^WT^ PM localization levels based on the quantitative 2A-assay system we developed. We predict these 64 mutants to be likely pathogenic due to DYSF protein misfolding leading to protein mislocalization, aggregation, and/or degradation. It is possible that other possible functions of dysferlin in muscle may remain intact in these mutants. We cannot conclude that the remaining 49 mutants above the 25% 2A-assay threshold are not pathogenic, because, even though they are PM localized, they may lack the ability to carry out muscle repair. Nevertheless, such *in vitro* characterizations of DYSF^PMMs^ are likely to aid clinicians in the diagnosis of individuals with dysferlinopathy through the (re)classification of PMMs as likely to be the cause of pathogenicity.

This assay may be amenable to high-throughput screening, thus allowing the identification of novel compounds to treat a subset of dysferlinopathies. As proof of principle, we show that the CFTR corrector, corr-2b, and the chemical chaperone 4-PBA can restore plasma membrane localization and damage repair activity to multiple mutant proteins.

4-PBA, known clinically as Buphenyl (sodium phenylbutyrate) or Ravicti (glycerol phenylbutyrate), was first approved in 1996 to treat patients with urea cycle disorders (Kolb et al., 2015). The metabolized drug complexes with toxic ammonia, creating an alternate ammonia elimination pathway in these patients. 4-PBA has also been frequently described as a chemical chaperone based on its ability to strongly attenuate ER stress and protein aggregate formation (Kolb et al., 2015; Luo et al., 2015; Mimori et al., 2013; Mizukami et al., 2010). More recently, 4-PBA combination therapy with taurursodiol showed positive findings in a clinical trial for ALS (Paganoni et al., 2020). We show that 4-PBA can restore localization to 25 of 64 DYSF^PMMs^ tested in our cell-based assay. These findings, along with the long record of 4-PBA safety in humans, suggests that 4-PBA could have a translational path to treat a subset of patients with dysferlinopathy. It would also be of interest to determine whether Dysferlin PM localization or membrane repair function declines with normal aging. If so, our platform may identify novel compounds to slow muscle loss in an aging population.

### Limitations of the study

One hundred thirteen DYSF missense patient mutations (PMMs) were analyzed in an HEK cell-based model system using live-cell flow cytometry to quantitatively determine the amount of PM-localized mutant DYSF protein present compared with cells expressing wild-type DYSF. Sixty-four DYSF PMMs were found to be PM localization defective in HEK cells. Several of these DYSF PMMs also failed to localize to the PM of myotubes generated from transfected dysferlin-deficient GREG mouse myoblasts. Although these results suggest that loss of DYSF at the PM is a possible mechanism of pathogenicity in patients harboring these mutations, conclusive determination would require analysis of DYSF expression and localization in patient samples. Furthermore, DYSF PMMs found at the PM in our assay system may be loss-of-function mutations and/or have localization deficiencies in patient cells/tissues that are not reflected in the *in vitro* assays described here and hence may have other mechanisms that produce pathogenicity.

The clinically approved drug, 4-phenylbutyric acid, was shown to restore PM-localization to 25 different DYSF PMMs in our *in vitro* 2A-assay system and restore membrane repair function following 2-day *in vivo* drug treatment in mice homozygous for DYSF^L1341P^. Follow-up studies examining 4-PBA drug dosage and longitudinal treatment will need to be performed in order to determine if 4-PBA affects dysferlinopathy onset and progression in mice using histological and physiological metrics. In addition, the ability of 4-PBA to restore sarcolemma localization and membrane repair function to the other localization-defective DYSF^PMMs^, apart from DYSF^L1341P^ and DYSF^R555W^, should be evaluated. Although 4-PBA is a clinically approved compound, further studies to determine the efficacy of the drug on other DYSF PMMs are required prior to commencement of clinical trials testing 4-PBA on patients with dysferlinopathy carrying such missense mutations.

## STAR★Methods

### Key resources table

**Table:** 

REAGENT or RESOURCE	SOURCE	IDENTIFIER
**Antibodies**		

Mouse anti-2A Peptide (3H4)	Novus Biologicals	Cat #: NBP2-59627
Mouse anti-NCL-Hamlet DYSF	Leica	Cat #: Hamlet-CE (Clone Ham1/7B6)
Rabbit anti-ROMEO DYSF (JAI-1-49-3)	abcam	Cat #: ab124684
Rabbit anti-TagRFP	Thermo Fisher Scientific	Cat #: R10367
Rabbit anti-GAPDH	abcam	Cat #: ab9485; RRID:AB_307275
Rabbit anti-sodium potassium ATPase (EP1845Y)	abcam	Cat #: ab76020; RRID:AB_1310695
Rabbit anti-Calreticulin (EPR3924)	abcam	Cat #: ab92516
Goat anti-Mouse IgG (H+L) (AF 488)	Thermo Fisher Scientific	Cat #: A11029; RRID:AB_2534088
Goat anti-Rabbit IgG (H+L) (AF 647)	Thermo Fisher Scientific	Cat #: A27040; RRID:AB_2536101
Goat anti-rabbit IgG (HRP)	Cell Signaling Technology	Cat #: 7074; RRID:AB_2099233
Horse anti-mouse IgG (HRP)	Cell Signaling Technology	Cat #: 7076; RRID:AB_330924

**Bacterial and virus strains**		

NEB® Stable E. coli	New England Biolabs	Cat #: C3040I

**Chemicals, peptides, and recombinant proteins**		

Dulbecco's Modified Eagle Medium (DMEM)	Genesee Scientific	Cat #: 25-501
Fetal Bovine Serum (FBS)	Avantor Seradigm	
Horse serum	GIBCO	Cat #: 16050130
Penicillin/ Streptomycin	Thermo Fisher Scientific	Cat #: 15140122
Primocin™	InvivoGen	Cat #: ant-pm-1
Chick embryo extract	Thermo Fisher Scientific	Cat #: MP92850145
Sodium pyruvate	Sigma-Aldrich	Cat #: S8636
Phosphate-buffered saline (PBS, pH 7.4)	Genesee Scientific	Cat #: 25-507
Trypsin-EDTA	Corning	Cat #: 25-053
Hanks' Balanced Salt Solution (HBSS)	Corning	Cat #: 14185052
Calcium chloride	Sigma-Aldrich	Cat #: C4901
HEPES	Sigma-Aldrich	Cat #: H3375
2x Gibson Master Mix	New England Biolabs	Cat #: E2611L
2x KAPA HiFi HotStart ReadyMix	Kapa BioSystems	Cat #: KK2602
Agarose	Sigma-Aldrich	Cat #: 2120-OP
Carbenicillin	Bio Basic	Cat #: 4800-94-6
ViaFect Transfection Reagent	Promega	Cat #: E4981
FM1-43 dye	Biotium	Cat #: 70020
4’ ,6-diamidino-2-phenylindole (DAPI)	BioLegend	Cat #: 422801
Mammalian Protein Extraction Reagent	Thermo Fisher Scientific	Cat #: 78501
PhosSTOP Phosphatase Inhibitor Cocktail Tablets	Roche	Cat #: 4906845001
cOmplete™, MiniProtease Inhibitor Cocktail Tablets	Roche	Cat #: 11697498001
Block Ace	BioRad	Cat #: BUF029
Bovine serum albumin (BSA)	Sigma-Aldrich	Cat #: A6003
ProLong™ Glass Antifade Mount	Thermo Fisher Scientific	Cat #: P36962
D-(+)-Glucose solution	Sigma-Aldrich	Cat #: G8769
Dimethyl sulfoxide (DMSO)	Sigma-Aldrich	Cat #: D2650
4-Phenyl butyric acid	Sigma-Aldrich	Cat #: P21005
sodium phenylbutyrate	Sigma-Aldrich	Cat #: SML0309
N-phenyl-4-(4-vinylphenyl)thiazol-2-amine	Exclusive Chemistry	Cat #: EC-000.2101
Collagenase	Sigma-Aldrich	Cat #: C0130
Doxycycline	Sigma-Aldrich	Cat #: D9891

**Critical commercial assays**		

Cell Titer 96™ AQueous Assay Kit	Promega	Cat #: G3582
QIAquick PCR Purification Kit	Qiagen	Cat #: 28104
Qiagen Gel Extraction Kit	Qiagen	Cat #: 28704
HiSpeed Plasmid Midi Kit	Qiagen	Cat #: 12643
Immun–Star^TM^ WesternC^TM^ Chemiluminescence Kit	BioRad	Cat #: 1705070

**Experimental models: Cell lines**		

Human: HEK293T cell	ATCC	
Mouse: GREG cell	Laboratory of Joshua Zimmerberg, NIH	

**Experimental models: Organisms/strains**		

Mouse: MMex38	Simone Spuler, Muscle Research Unit Experimental and Clinical Research Center – a joint cooperation of the Charité Medical Faculty and the Max Delbrück Center for Molecular Medicine Berlin, Germany	N/A
Mouse: C57BL/6NJ	Jackson Laboratories	Cat#: 005304

**Oligonucleotides**		

Human: *DYSF*^*WT*^ Forward 5’- CTG ACG CGT TTG GTT ATG CAA TGG ATT ACA AGG ATG ACG ATA AG -3’	This paper	N/A
Human: *DYSF*^*WT*^ Reverse 5’- CCT CTG CCC TCT GGC ATG CAG CTG AAG GGC TTC ACC AG -3’	This paper	N/A
See Table S1 for oligonucleotides used for generating mutant DYSF constructs	Table S1	

**Recombinant DNA**		

LV-TRE-VP64 human MyoD-T2A-dsRedExpress2	Kabadi et al. (2015)	Addgene plasmid #60629
pCI FLAG-hDysferlin	Laboratory of Robert Brown, UMass	N/A

**Software and algorithms**		

R software (v3.4.4)	R Core Team	https://www.r-project.org
Fiji	Schindelin et al. (2012)	https://imagej.net/software/fiji/
Flowing Software 2	Cell Imaging Core at the Turku Centre for Biotechnology	https://bioscience.fi/services/cell-imaging/flowing-software/

**Other**		

Transnetyx® genotyping service	Transnetyx	https://www.transnetyx.com

### Resource availability

#### Lead contact

Further information and requests for resources and reagents should be directed to and will be fulfilled by the lead contact, Dr. Leonard P. Guarente (leng@mit.edu)

#### Materials availability

Plasmids generated in this study are available through the lead contact; however, investigators should check if the plasmid(s) are available through Addgene first.

### Experimental model and subject details

#### Cell lines

Human (female) embryonic kidney cells, HEK293T, were purchased from the American Type Culture Collection. HEK cells were cultured in Dulbecco's Modified Eagle Medium (DMEM; Genesee Scientific) containing 10% Fetal Bovine Serum (FBS, Avantor Seradigm), 100 units/mL penicillin, and 100 μg/mL streptomycin (Thermo Fisher Scientific); culture media was changed every two days. HEK cells were grown to sub confluence at 37°C in a humidified 5% CO_2_ incubator and passaged at a ratio of 1:4 - 1:8. Western blot analysis using Hamlet α-DYSF Ab authenticated that HEK cells do not express endogenous dysferlin protein under these culture conditions.

GREG cells, a DYSF-deficient myogenic cell line established from the A/J mouse strain (Humphrey et al., 2012) (sex of animal used to establish cell line were not reported), were cultured in DMEM/Glutamax (ThermoFisher Scientific) containing 20% FBS, 1 mM sodium pyruvate (Sigma), 0.5% chick embryo extract (Thermo Fisher Scientific), and 100 μg/mL Primocin™ (InvivoGen). Cells were washed with PBS and cultured in DMEM + 5% horse serum, 1 mM sodium pyruvate (Sigma), and 100 μg/mL Primocin (InvivoGen) to induce cell fusion and myotube differentiation; media was changed daily. GREG cells were grown to 50% confluence at 37°C in a humidified 5% CO_2_ incubator and passaged at a ratio of 1:4.

#### MMex38 mice

MMex38 mice were obtained from Max Delbrück Center, Berlin (Malcher et al., 2018). C57BL/6NJ wild type mice purchased from Jackson Laboratories. All animal husbandry, care, and procedures were performed according to a Massachusetts Institute of Technology Committee on Animal Care IACUC-approved protocol. Mice were housed in a temperature-controlled facility (25 ± 1°C) with a 12-h:12-h light-dark cycle and fed standard rodent chow diet.

MMex38 animals were rederived by embryo implantation and housed in the MIT mouse facilities. MMex38 mice were backcrossed once to their progenitor strain C57BL/6NJ and homozygous DYSF^L1360P^/DYSF^L1360P^ and +/+ lines were established from sibling F2 progenies. Experiments were performed with age-matched siblings of the same gender for each genetic line. Mouse genotyping was performed by Transnetyx® genotyping service using primer pairs and genotyping protocol described for MMex38 male mice (Malcher et al., 2018).

#### 4-PBA treatment

Sodium phenyl butyrate (USP, Sigma) was dissolved in sterile drinking water at a concentration of 2 mg/mL. C57BL6/NJ (+/+) and MMex38 male mice, aged 16, 20, and 38 weeks were treated ad-libitum for 48-hrs either with drug or water alone. Age-matched mice were used in each experiment. Following treatment, mice were euthanized, and EDL muscle isolated for subsequent use in membrane repair assays and fresh frozen sections prepared for histological analysis.

### Method details

#### Dysferlin (DYSF) expression vectors

LV-TRE-DYSF-T2A-DsRed was constructed by Gibson Assembly using plasmids LV-TRE-VP64 human MyoD-T2A-dsRedExpress2, a gift from Charles Gersbach (Addgene plasmid #60629) (Kabadi et al., 2015) and pCI FLAG-hDysferlin (gift from Dr. Robert Brown, University of Massachusetts, Worcester), containing human dysferlin cDNA (isoform 8, NCBI accession NM_003494) with an N-terminal 18 amino acid flag-tag-containing sequence. Digestion of LV-TRE-VP64 human MyoD-T2A-dsRedExpress2 with NsiI excises the VP64 human MyoD cassette, leaving a 10kB vector backbone. This Nsi fragment was replaced with a 6,334 bp PCR product containing hDYSF^WT^ cDNA, generated with DYSF^WT^ F-(5’CTG ACG CGT TTG GTT ATG CAA TGG ATT ACA AGG ATG ACG ATA AG 3’) and DYSF^WT^ R- (5’CCT CTG CCC TCT GGC ATG CAG CTG AAG GGC TTC ACC AG 3’) primers using pCI as template. Gibson assembly of these two fragments generated the 16,301 bp LV-TRE-DYSF^WT^-T2A-DsRed bicistronic expression vector regulated by a minimal CMV promoter and tetracycline response element (Figure 1A); plasmid sequencing verified proper assembly and DYSF^WT^ sequence.

LV-TRE-DYSF^PMM^-T2A-DsRed expression vectors, listed in Table S2, were subsequently generated according to the scheme in Figure S2. Briefly, DYSF PMMs were introduced into each of two products via the PCR1^PMM^ Reverse primer or PCR2^PMM^ Forward primer used for PCR synthesis (Table S1) using LV-TRE-DYSF^WT^-T2A-DsRed as the PCR DNA template. The PCR1^PMM^ Forward primer and PCR2^PMM^ Reverse primers contain vector homology encompassing each restriction site necessary for Gibson assembly. The choice of restriction sites utilized for Gibson cloning was dependent upon the location of the PMM within DYSF (Table S1). PCR1^PMM^ and PCR2^PMM^ (Figure S2) products were generated in 25 μL reactions with 5 ng pCI template in 10 PCR cycles (95°C 15 sec, 60°C 30 sec, 72°C 2’) using the primer pairs listed in Table S1 and Kapa HiFi HotStart PCR Master Mix (Roche). For any given PMM, the two corresponding PCR reactions were combined and purified using a PCR purification kit (Qiagen) and eluted in 30 μL. These combined products were then subject to PCR amplification (95°C 20 sec, 72°C 3’) with Kapa HiFi DNA polymerase without primers for 5 cycles to allow products to anneal and extend, creating a PCR stitched product. Subsequently, 0.3 pM of both PCR1 Forward and PCR2 Reverse primers (10pM/μL) were then added and the reaction was amplified for 10 additional cycles (95°C 15 sec, 60°C 30 sec, 72°C 3’), amplifying the stitched product. Reactions were run on a 1% agarose gel, and full length bands excised and purified using an agarose gel purification kit (Qiagen). DYSF^PMMs^ listed in Table S1were introduced into LV-TRE-DYSF^WT^-T2A-DsRed by Gibson assembly of the stitched and amplified PMM-containing PCR product with gel purified linear LV-TRE-DYSF^WT^-T2A-DsRed vector DNA digested with specific restriction enzymes for each PMM (Figure S1, Table S1). Gibson assemblies were performed in 10 μL 1x Gibson Assembly® Master Mix (NEB) with 100 ng of restriction enzyme digested, gel-purified linearized vector and approximately 10-20 ng of purified PCR product. Reactions were incubated at 50°C for 6 hours prior to 1-hr drop dialysis with water using a 0.025 μM type-VS membrane filter (Millipore, Inc. #VSWP 02500) and subsequent electroporation into NEB® Stable *E. coli* followed by selection and growth at 30°C on LB agar + carbenicillin (100 μg/mL) plates. Clones were screened for assembly and the PCR amplified regions fully sequenced with overlapping primers to verify the presence of PMM with no PCR amplification errors. DNA for transfection was prepared by electroporation of plasmid into NEB Stable cells, overnight growth at 30°C in LB liquid + carbenicillin (100 μg/μL), and DNA purification using HiSpeed Plasmid Midi Kit (Qiagen).

#### Transient transfections

HEK cells were seeded at a density of 6 × 10^5^ cells/well and GREG cells at 3 × 10^5^ cells/well in 6-well plates and incubated for 24-hrs. HEK cells were transfected with plasmid DNA (1.5 μg) using calcium dichloride (120 μmol/mL, Sigma) in HEPES buffer (Sigma) with media change after 4hr of incubation, and collection 48-hrs later for flow cytometry. GREG cells were transfected with plasmid DNA (1.5 μg) and ViaFect Transfection Reagent (E4981, Promega, USA) using the 3:1 low-volume manufacturer’s protocol with subsequent culture in 2 μg/mL doxycycline. Based on flow cytometry detection of DsRed the typical transfection efficiency of was 20% for HEK cells and 5-10% for GREG cells.

#### Cell viability/proliferation assay

HEK cells were seeded in 96-well plates at 5,000 cells/well, After 24-hrs, media was supplemented with compounds or DMSO (Sigma) vehicle control and incubated for 24-hrs before cell viability was measured using Cell Titer 96™ AQ_ueous_ Assay Kit (Promega) according to the manufacturer’s specifications.

#### 2A-assay

Transfected HEK cells were trypsinized and placed on ice at 4°C for 10 min and then incubated with α-2A antibody (Ab) (1:250 dilution, clone:3H4, Novus Biological) in DMEM containing 2% FBS at 4°C for 30 min. Cells were washed with ice-cold PBS and incubated with the secondary Ab Alexa 647 α−mouse IgG (1:250, Thermo Scientific) at 4°C for 30 min. Cells were subsequently washed with cold PBS and treated with membrane impermeant DAPI (BioLegend) to stain dead cells. Live cells were analyzed by flow cytometry using a Fortessa FACS (BD Bioscience), and the flow data was analyzed using Flowing Software 2 (Cell Imaging Core, Turku Centre for Biotechnology). 2A-expression values for any given DYSF^PMM^ are reported relative to DYSF^WT^ and is a ratio of the calculated **g**eometric **m**ean **f**luorescence **i**ntensity (gMFI) of Alexa-647 that detects the extracellular DYSF-2A epitope relative to the gMFI of intracellular DsRed from the same group of live (DAPI negative) cells. 2A-assay values were calculated as follows: (gMFI Alexa-647 HEK-DYSF^PMM^/gMFI DsRed HEK-DYSF^PMM^)/ (gMFI Alexa-647 HEK-DYSF^WT^/gMFI DsRed HEK-DYSF^WT^), with reported means and standard deviations from at least three independent experiments.

For drug treatment analysis, transiently transfected HEK cells were plated a density of 5 × 10^4^ cells/well into 96-well plates. After incubation for 24-hrs, compounds were added at indicated concentration in DMEM with 2% FBS, and incubated for a further 24-hr . Cells were trypsinized for 1 min and Ab treatments were carried out as above in 96-well U-bottom plates prior to flow cytometry analysis.

#### Western blot

Cultured cells were washed with PBS and lysed with Mammalian Protein Extraction Reagent (M-PER, Thermo Scientific) with the phosphatase inhibitor cocktail (PhosSTOP, Roche) and the protease inhibitor cocktail (cOmplete, Roche). Protein samples were separated on a 4-15% SDS-PAGE gel (TGX gel, Bio-Rad) and transferred to polyvinylidene fluoride (PVDF) membrane. Membranes were incubated in Block Ace (BioRad) at 4°C overnight and then with anti-NCL-Hamlet DYSF (mouse, 1:2,000, Leica), anti-RFP (DsRED, rabbit, 1:1,000, Thermo Scientific) and anti-GAPDH (rabbit, 1:5,000, Abcam) as primary monoclonal antibodies. Membranes were washed three times in PBS with 0.1% Tween-20, then with horseradish peroxidase (HRP)-conjugated anti-rabbit IgG (1:5,000, Cell Signaling Technology) or anti-mouse IgG (1:10,000, GE Healthcare Life Science) as secondary antibodies. All antibody incubations were performed at room temperature (RT) for 1-hr. Blots were developed using Immun–Star™ WesternC™ Chemiluminescence Kit (BioRad) and imaged using an A600 imager (Azure Biosystems). Band intensities were analyzed using Fiji (Fiji Is Just ImageJ, (Schindelin et al., 2012)) image processing software.

#### Immunofluorescence (IF)

Transfected DsRed positive sorted GREG cells and EDL muscle sections were fixed in pre-chilled ethanol for 10 min at −20°C, washed in PBS containing 0.1% Triton X-100 for 10 min, blocked with 3% bovine serum albumin (BSA, Sigma) at RT for 1-hr, and incubated at RT for 1-hr with one or more of the following primary antibodies: anti-NCL-Hamlet DYSF (1:200), anti-Romeo DYSF (1:200), anti-sodium potassium ATPase (1:250, EP1845Y, Abcam) for plasma membrane localization, anti-calreticulin (EPR3924, Abcam) for ER localization, and anti-2A peptide. After wash with PBS, cells were incubated with Alexa Fluor 488 anti-mouse IgG (Thermo Scientific) and Alexa Fluor 647 anti-rabbit IgG (Thermo Scientific) as secondary antibodies at RT for 1-hr. Coverslips were enclosed using ProLong™ Glass Antifade Mount (Thermo Fisher Scientific) after DAPI treatment at RT for 5 min. Images were obtained with a fluorescent microscope (Carl Zeiss).

#### Membrane repair assays

Plasma membrane repair capacity of GREG myotubes and myofibers were measured by the kinetics of intracellular uptake of the normally membrane impermeant dye FM1-43 following membrane laser wounding. DsRed positive GREG myoblasts are sorted by flow cytometry (FACS Aria) and seeded in a chambered cover glass (Nunc™ Lab-Tek™ II 155409PK, Thermo Scientific, USA). After an hour, culture media is changed to differentiation medium and incubated for 48-hrs to obtain myotubes. Prior to assay, the culture media is removed and switched to PBS with 1 mM calcium dichloride, glucose (4 mg/mL), and FM1-43 (5 ng/μL, Biotium).

EDL muscle was surgically isolated from euthanized MMex38 or C57BL/6NJ male mice of specified ages and placed in solution, treated with 0.2% collagenase type I (Sigma-Aldrich) for 1.5-hrs at 37°C and then pipetted to disrupt the tissue, and then put into a culture dish with culture medium and 4-PBA (1 mM) or DMSO for 24-hrs.

Membrane repair assays and imaging are performed at RT with the chambered cover glass mounted on a confocal microscope (Olympus FV1200, 63x, 0.9 NA, oil immersion objective with laser scanning at 488 nm). To laser wound the myotubes, a region on the PM was irradiated with a 405 nm laser at 60-100% power (15 mW) using the photo-activation mode for 5-10 seconds, depending upon myotube treatment conditions. Imaging begins prior to membrane wounding with each frame separated by 1.1 seconds, continuing for approximately 3 minutes. Image analysis of dye uptake in an intracellular region of interest (ROI) adjacent to the site of laser injury was obtained using Fiji. Net change in ROI fluorescence for any image frame following wounding (ΔF) was calculated by subtracting the background fluorescence in the ROI at the moment before wounding (F_0_) is plotted for every 10th frame (11 seconds) after wounding in visualize relative membrane repair capacities.

### Quantification and statistical analysis

Statistical analysis was performed using R software (v3.4.4). Student’s unpaired t-test was used to compare differences between two samples, and values of ∗p < 0.05, ∗∗p<0,01, ∗∗∗p<0.001 were considered significant. Values are presented as means ± S.D.

## Data Availability

This paper does not report original code. All data reported in this paper will be shared by the lead investigator upon request. Any additional information required to reanalyze the data reported in this paper is available from the lead contact upon request.
