# Supplementary material for: 4-Phenylbutyrate restores localization and membrane repair to human dysferlin mutations
Source: iScience. 2021 Dec 20;25(1):103667. doi: 10.1016/j.isci.2021.103667 (PMC8741482; doi:10.1016/j.isci.2021.103667)
Supplement: Document S1. Figures S1–S12 and Tables S1–S3 [file mmc1.pdf]

iScience, Volume *25*

Supplemental information

4-Phenylbutyrate restores localization and membrane repair to human dysferlin mutations

Kana Tominaga, Naoomi Tominaga, Eric O. Williams, Laura Rufibach, Verena Schöwel, Simone Spuler, Mohan Viswanathan, and Leonard P. Guarente

Supplementary Figures

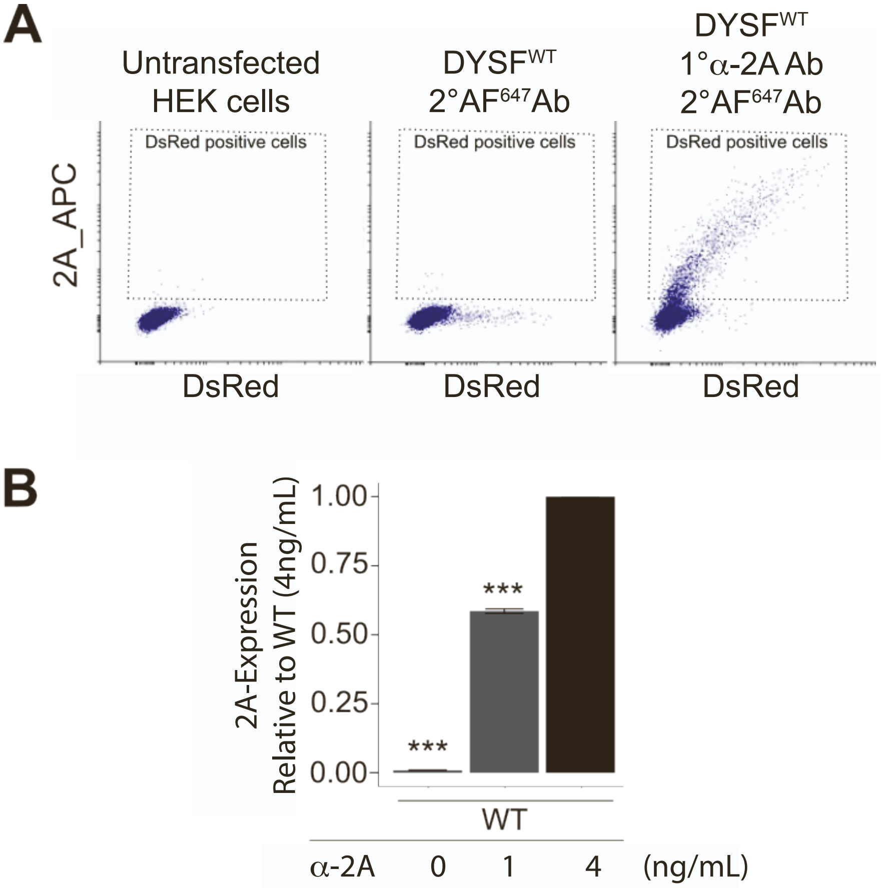

Fig. S1. Validation of α-2A Ab detection of PM-localized DYSF in 2A assay, related to Figure 1. (A) Flow cytometry dot plots analyzing DYSF-2A detection in non-transfected HEK cells and HEK cells transiently transfected with $DYSF^{WT}$. Cells expressing $DYSF^{WT}$ were hybridized with α-2A 1˚ Ab and Alexa Fluor 647 conjugated-IgG 2˚Ab as labelled. The flow cytometer fluorescence wavelength detector was set for allophycocyanin (APC), which detects Alexa Fluor 647. **(B)** Determination of α-2A 1˚ Ab range of detection of PM-localized $DYSF^{WT}$ in HEK cells (n = 3). *** $p < 0.001$ indicates difference between 4 ng/ml and 0 or 1 ng/ml.

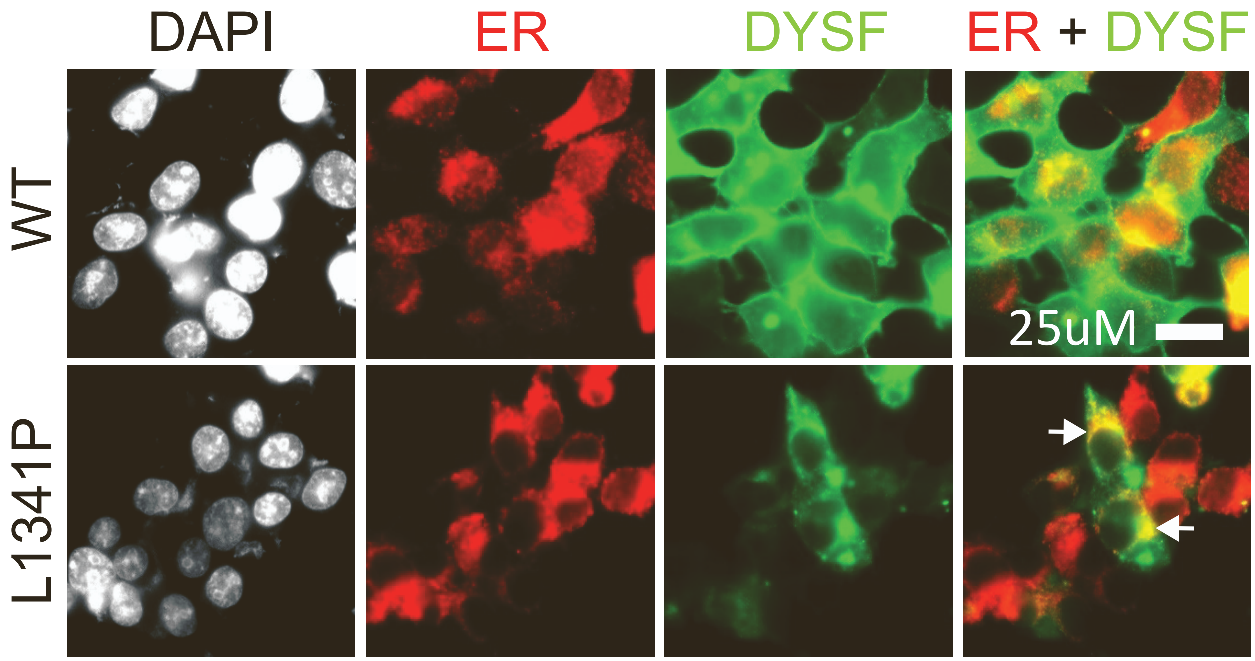

Fig. S2. Immunofluorescence images localizing DYSF^{PMMs} expressed in HEK293T cells, related to Figure 1. 48-hrs post transfection HEK293T cells expressing DYSF^{L1341P} were FACS sorted for DsRed, cultured, fixed, and hybridized with the Hamlet α-DYSF 1˚ Ab (green) and the α-Calreticulin 1˚ Ab (red) to identify the endoplasmic reticulum (ER). Arrows point to perinuclear areas where DYSF^{L1341P} localization is coincident with ER. Scale bar: 25 microns.

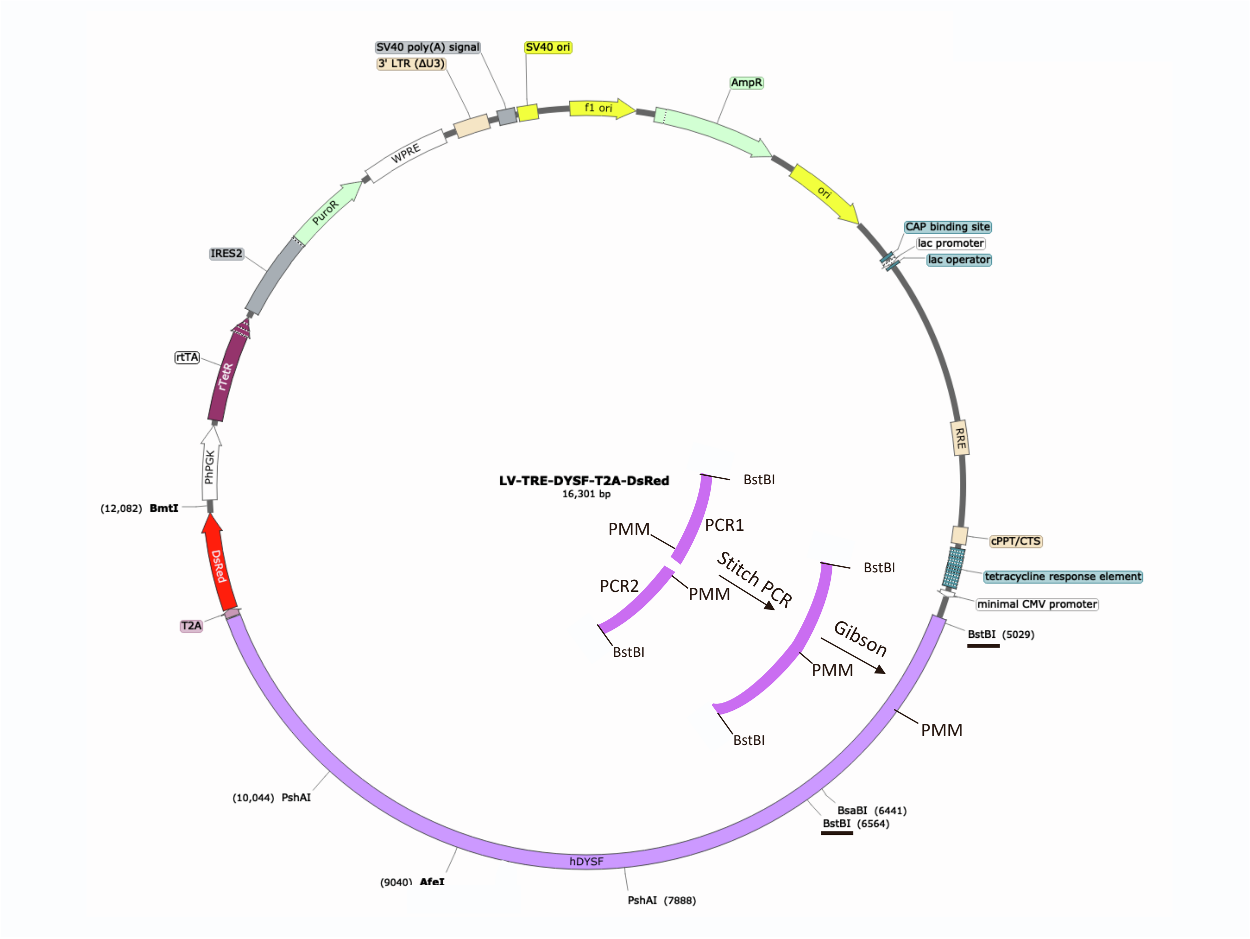

Fig. S3. PCR stitching and Gibson cloning scheme for introduction of DYSF PMMs into LV-TRE-DYSF-T2A-DsRed, related to Figure 1 and Table S1. A DYSF^{PMM}-containing PCR product was produced by PCR stitching two separate PCR products, PCR1 and PCR2, each with overlapping regions of homology containing the PMM introduced in the PCR oligonucleotides. This product was then used for Gibson assembly into linearized LV-TRE-DYSF-T2A-DsRed using the appropriate restriction enzymes listed in Table S1. Example shown is for Gibson assembly of PCR products containing DYSF^{PMMs} between BstBI sites, similar products were generated for other regions of DYSF.

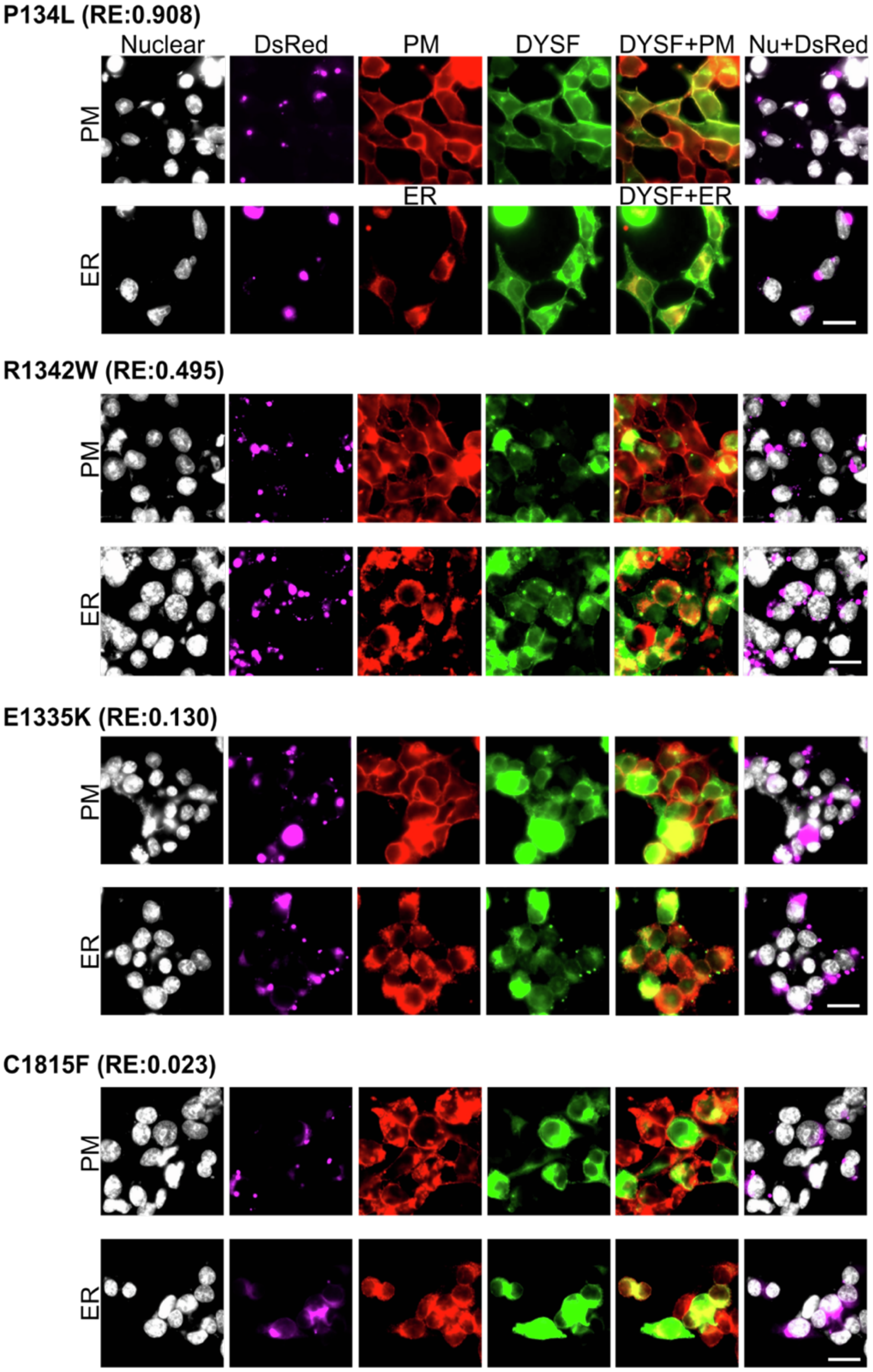

Fig. S4. Immunofluorescence images showing PM localization of DYSF in HEK cells expressing DYSF^{P134L}, DYSF^{R1342W}, DYSF^{E1335K}, or DYSF^{C1815F}, related to Figure 2. HEK cells were transiently transfected with a DYSF^{PMM} expression construct, cultured for 48-hrs, and sorted for DsRed positive cells. Following 24-hrs of culture, cells were processed for ICC with α-DYSF Ab (green), α-sodium potassium ATPase Ab PM (red) and α-calreticulin Ab ER (red) to aid in identification of these organelles, as well as DAPI nuclear marker (Nu). RE, Relative expression to WT. Scale bar: 25 microns.

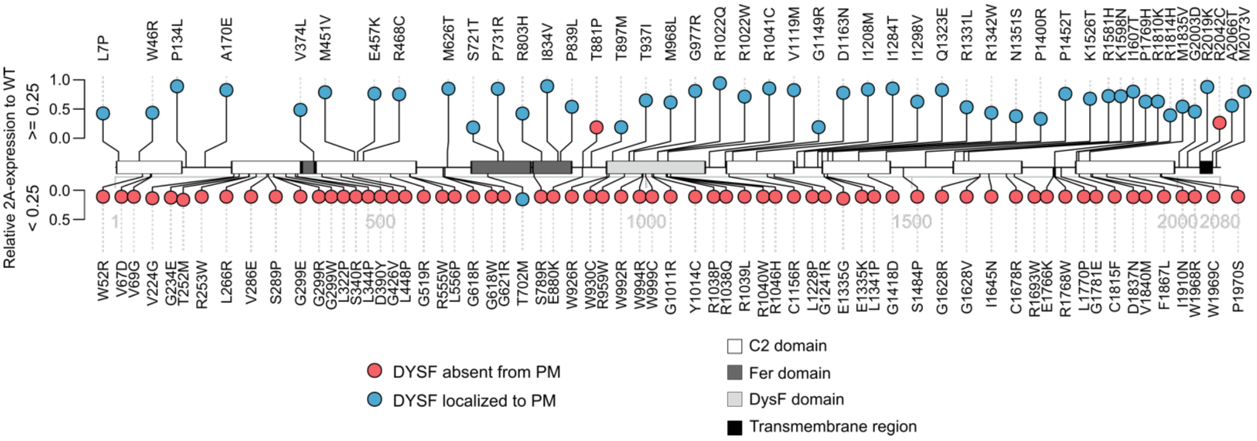

Fig. S5. 2A-assay and ICC results for 113 DYSF^{PMMs} expressed in HEK cells: DYSF gene lolliplot, related to Figure 2 and Table S1. Plotted on the y-axis (DYSF protein domains annotated) are the average 2A-assay values (n=3) of PM-localized DYSF^{PMM} relative to DYSF^{WT}. Values are directionally plotted based on 25% threshold of PM-localization in 2A-assay with PMMs below threshold plotted below the x-axis in red and PMMs above threshold plotted above the x-axis in blue. Lolliplot was created using R Bioconductor.

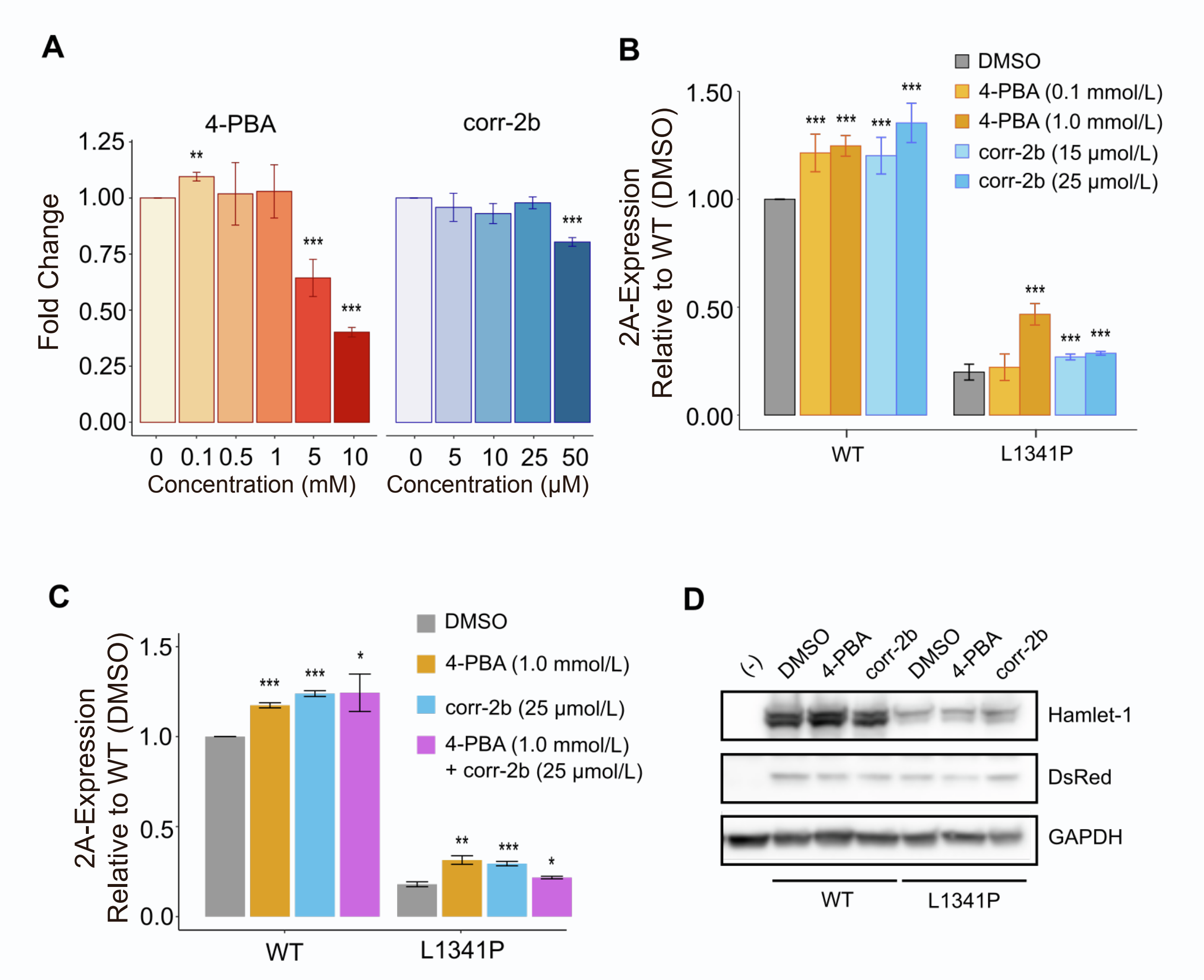

Fig. S6. Evaluation of 4-PBA and corr-2b efficacy, related to Figure 3. **(A)** Cell proliferation assays for HEK cells treated with 4-PBA or corr-2b compared to DMSO alone for 24-hrs demonstrates that 4-PBA and corr-2b prevent cell proliferation at concentrations greater than 1mM and 25 µM, respectively **(B)** Dose response of 4-PBA and corr-2b in 2A-assays with DYSF^{L1341P} shows optimal effect of 4-PBA and corr-2b are at 1mM and 25uM, respectively. All values are normalized to cells expressing DYSF^{WT} treated with DMSO. **(C)** Combination of 4-PBA and corr-2b treatment shows no additivity compared with a single treatment in 2A-assay response relative to DMSO treated DYSF^{WT}. **(D)** Effect of drug treatments on DYSF expression in HEK cells transiently transfected with DYSF^{WT} or DYSF^{L1341P}. HEK cells expressing DYSF^{WT} or DYSF^{L1341P} were incubated with either DMSO (0.1%), 4-PBA (1 mM), corr-2b (25 µM) for 24-hrs and subject to western blot of total protein lysates from each cell line under each treatment. Non-transfected HEK cells were used as negative control. Membranes were hybridized with α-DYSF(Hamlet) Ab, and α-DsRed 1˚ Ab and α-GAPDH 1˚ Ab as loading control. *p < 0.05, **p<0,01, ***p<0.001, relative to untreated or DMSO control.

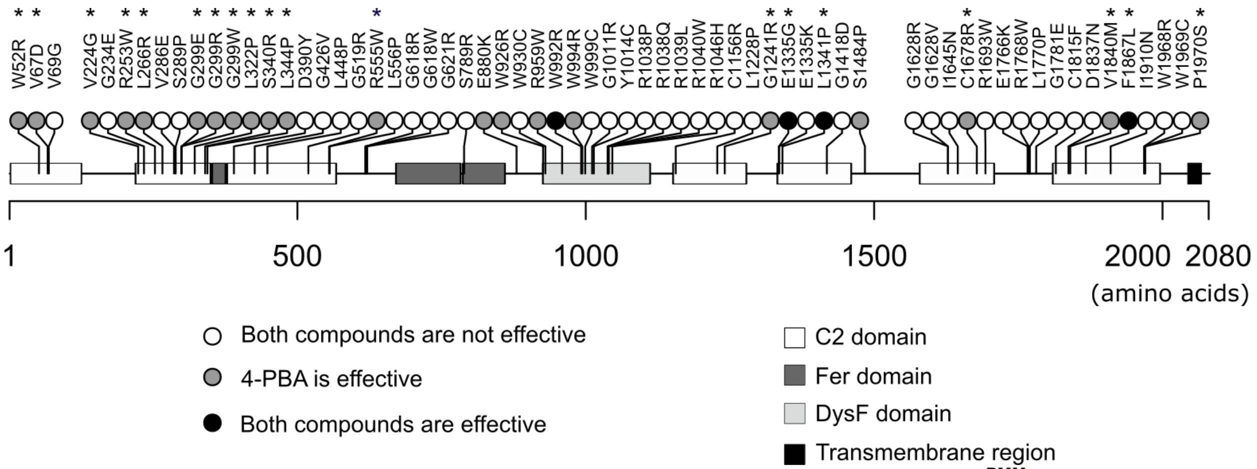

Fig. S7. 4-PBA is highly effective in restoring PM-localization to various DYSF^{PMMs} across the various domains, related to Figure 3. Lolliplot showing effect of 4-PBA or corr-2b treatment on 64 missense mutations across the *DYSF* cDNA. Asterisks show nineteen mutations in C2 domains rescued by 4-PBA. Lolliplot was created using R Bioconductor.

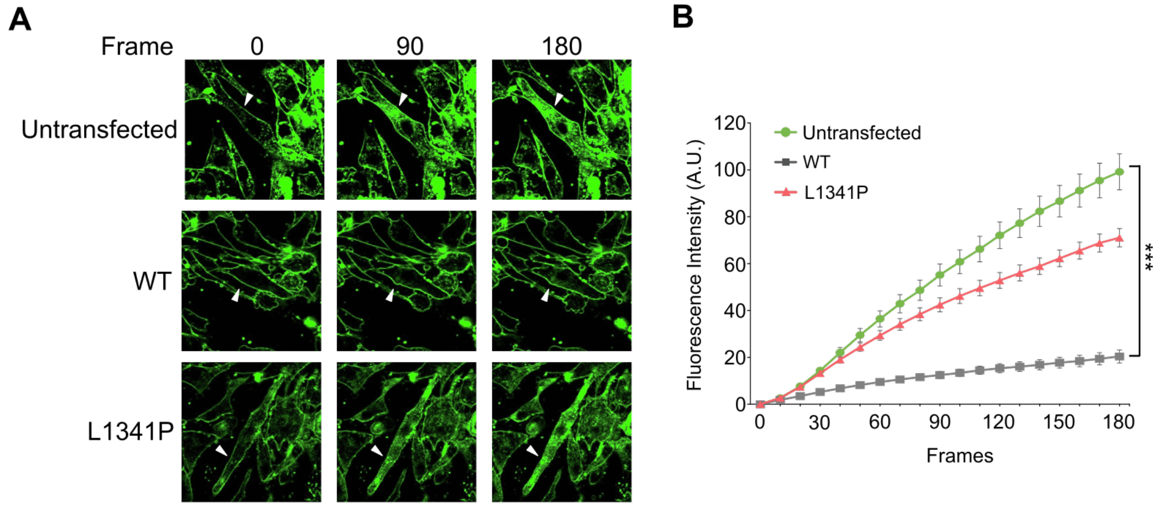

Fig. S8. Membrane repair in GREG cell derived myotubes, related to Figures 2, 4A, 4B, and S9. Dysferlin deficient GREG myoblasts were transfected with DYSF^{WT} or DYSF^{L1341P} expression vectors, cultured, and subsequently sorted for DsRed positive myoblasts. Sorted cells, as well as non-transfected GREG cells, were plated in chambered cover glass in differentiation media and cultured into myotubes. Myotubes were laser (405nm) wounded in the presence of calcium and FM1-43 dye (green). Untransfected and DYSF^{L1341P} transfected myotubes failed to repair laser induced membrane damage, while myotubes expressing DYSF^{WT} rapidly repaired the breach. **(A)** Three image frames are presented for each sample; the first image is at the time of wounding, the second in the middle of repair, and the third at the end of repair. Arrowheads show the site of injury. **(B)** Repair kinetics of non-transfected GREG cells (n = 13), and GREG cells expressing DYSF^{WT} (n = 19) or DYSF^{L1341P} (n = 23) as measured by FM1-43 dye influx into the myotube injury site. Data area means +/- S. E. M. ***$p<0.001$

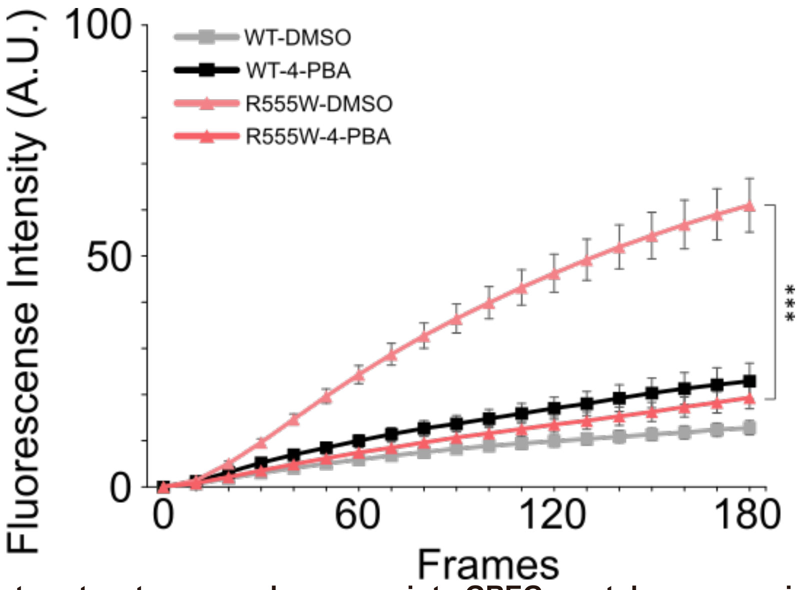

Fig. S9. 4-PBA treatment restores membrane repair to GREG myotubes expressing $DYSF^{R555W}$, related to Figures 3A, 4A, and 4B. Kinetic analysis of FM1-43 dye influx in transfected dysferlin deficient GREG myotubes after laser membrane damage. Number of transfected and treated myotubes assayed: $DYSF^{WT}$(DMSO) (n = 10), $DYSF^{WT}$ (4-PBA) (n = 10), $DYSF^{R555W}$(DMSO) (n = 15), or $DYSF^{R555W}$ (4-PBA) (n = 13). Data are means ± S.E.M, ***p<0.001.

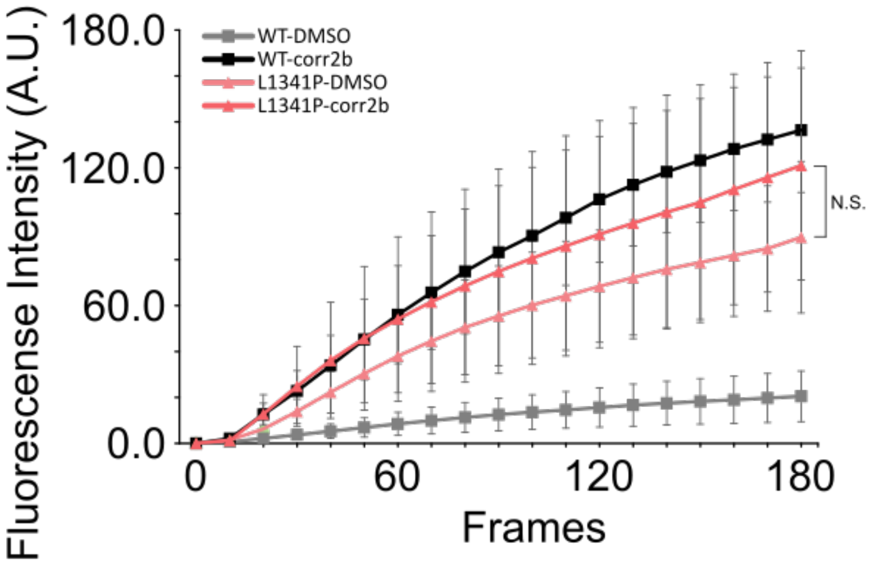

Fig. S10. corr-2b treatment of GREG myotubes prevents membrane repair in the presence of $DYSF^{WT}$ and sensitizes non-transfected GREG myotubes to damage, related to Figure 3. Kinetic analysis of FM1-43 dye influx in dysferlin deficient GREG myotubes after laser damage (405nm). Number of transfected and treated myotubes assayed: $DYSF^{WT}$(DMSO) (n = 12), $DYSF^{WT}$ (corr-2b) (n = 6), $DYSF^{L1341P}$ (DMSO) (n = 9), or $DYSF^{L1341P}$ (corr-2b) (n = 7). N.S.; not significant. Plotted values are means ± S.E.M.

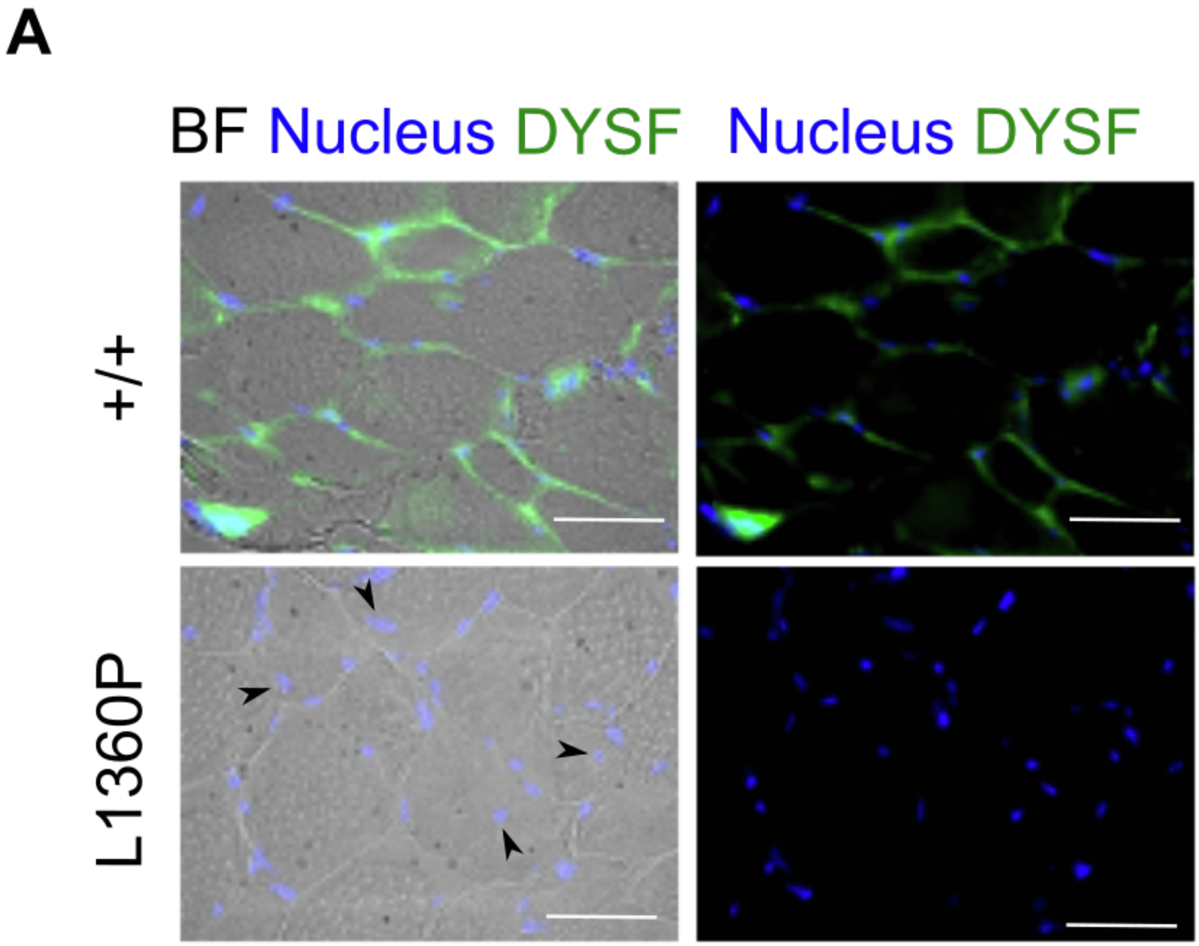

Fig. S11. Histological analysis of EDL muscle cross-section from MMex38 DYSF^{L1360P} mice show hallmarks of dysferlinopathy, related to Figures 4C, 4D, and 4E. Brightfield and immunofluorescence images of EDL muscle fibers obtained from C57BL/6NJ (20-month, male) or MMex38 (20-month, male) mice using Romeo α-DYSF 1˚ Ab with DAPI nuclear staining shows that sarcolemma localization of DYSF seen in control animals is absent in MMex38, as well as central nuclei (arrows) within myofibers in MMex38 mice, indicative of dystrophic muscle. Scale bar: 50 microns.

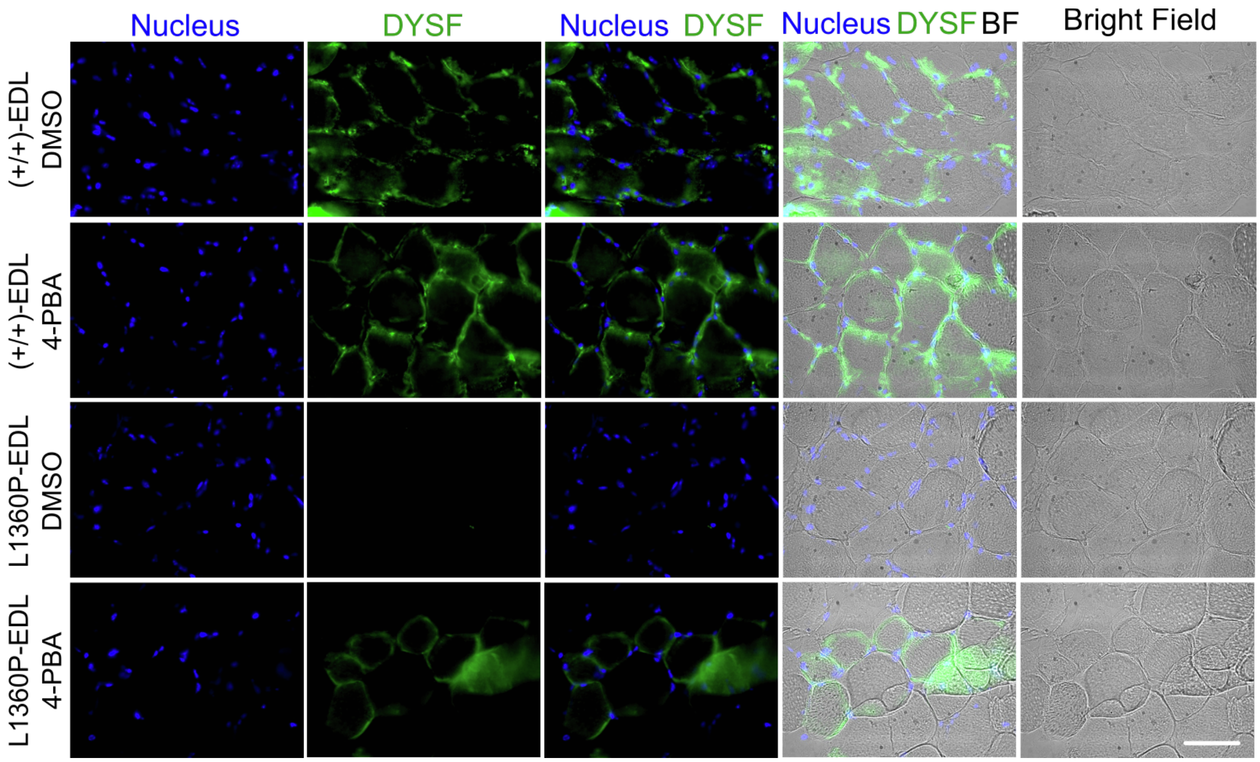

Fig. S12. *Ex vivo* treatment of MMex38 mice EDL with 4-PBA rescues DYSF sarcolemma expression, related to Figure 4. EDL muscle isolated from male C57BL6/NJ (+/+) and MMex38 mice and cultured in either vehicle (0.1% DMSO) or 4-PBA (1mM) for 24-hrs.
Fresh frozen cross-sections of the treated EDL muscles were subsequently prepared for histological analysis. DAPI staining was performed for nuclear localization and DYSF staining was done using Hamlet α-DYSF 1º Ab and Alexa 647 α-mouse fluorescent 2º Ab. Images were all taken at the same exposure time and magnification. Scale bar: 50 microns. Brightfield images and overlays show myofiber structures in the absence of any staining. Muscle cross section of EDL muscle from untreated MMex38 mice show no DYSF expression, while similar cross sections from 4-PBA treated MMex38 mice, shows increased $DYSF^{L1360P}$ expression and staining of myofiber sarcolemma.

Supplemental Tables

Table S1:DYSF^{PMMs} Engineering and HEK Cell Results										
PMM			Assay Result			Oligonucleotides used for generating LV-TRE-DYSFPMMs-T2A-DsRed				
			2A		ICC	PCR1		PCR2		
Clone #	Missense Mutation	Protein Change	Mean	S.D.	DYSF PM Localized?	F1	R1	F2	R2	Vector Digestion Enzyme(s)
1	20 T>C	L7P	0.486	0.016	Yes	tacaaggatgacgataagttcgaaCTCGAGCCCGGGGAATTC	ttctcggcatagGGGATGAAGACCCTCAGG	tcttcatcccCTATGCCGAGAACGTCCAC	tcatccaagtctgaggctttcgaaGGCTTGACAGCACCTGCAG	BstBI
2	136T>C	W46R	0.496	0.033	Yes	tacaaggatgacgataagttcgaaCTCGAGCCCGGGGAATTC	ccctcattccGTACAGGGTTCACGCTGTTC	aaccctgtacGGAATGAGGGATTTGAATGGGAC	tcatccaagtctgaggctttcgaaGGCTTGACAGCACCTGCAG	BstBI
3	154T>C	W52R	0.140	0.011	No	tacaaggatgacgataagttcgaaCTCGAGCCCGGGGAATTC	ttgaggtcccGTTCAAATCCCTCATTCCATACAGG	ggatttgaacGGGACCTCAAGGGCATCC	tcatccaagtctgaggctttcgaaGGCTTGACAGCACCTGCAG	BstBI
4	200TG>AT	V67D	0.146	0.018	No	tacaaggatgacgataagttcgaaCTCGAGCCCGGGGAATTC	ctttgaccacATCATGAAGCTCAGAGCCCTGG	gcttcatgatGTGGTCAAAGACCATGAGAC	tcatccaagtctgaggctttcgaaGGCTTGACAGCACCTGCAG	BstBI
5	206T>G	V69G	0.157	0.018	No	tacaaggatgacgataagttcgaaCTCGAGCCCGGGGAATTC	atggtctttgCCCACCACATGAAGCTCAG	atgtggtgggCAAAGACCATGAGACGATGGG	tcatccaagtctgaggctttcgaaGGCTTGACAGCACCTGCAG	BstBI
6	401C>T	P134L	0.908	0.067	Yes	tacaaggatgacgataagttcgaaCTCGAGCCCGGGGAATTC	agtagggggcAGGAACAGGGGCACAGCTC	ccctgttcctGCCCCCTACTCCTCTGGAG	tcatccaagtctgaggctttcgaaGGCTTGACAGCACCTGCAG	BstBI
7	509C>A	A170E	0.850	0.077	Yes	tacaaggatgacgataagttcgaaCTCGAGCCCGGGGAATTC	gaatggctccTCCTCATCTCCAGTGAGTCC	gagatgaggaGGAGCCATTCCTGGATCAAAG	tcatccaagtctgaggctttcgaaGGCTTGACAGCACCTGCAG	BstBI
8	671T>G	V224G	0.224	0.016	No	tacaaggatgacgataagttcgaaCTCGAGCCCGGGGAATTC	gatcacctggCCCCTGATCTGGAAATCCTG	agatcaggggCCAGGTGATCGAGGGGCG	tcatccaagtctgaggctttcgaaGGCTTGACAGCACCTGCAG	BstBI
9	701G>A	G234E	0.213	0.019	No	tacaaggatgacgataagttcgaaCTCGAGCCCGGGGAATTC	gatgttcaccTCCGGCAGCTGGCGCCCC	agctgccggaGGTGAACATCAAGCCTGTGG	tcatccaagtctgaggctttcgaaGGCTTGACAGCACCTGCAG	BstBI
10	755C>T	T252M	0.248	0.039	No	tacaaggatgacgataagttcgaaCTCGAGCCCGGGGAATTC	gtggatccgcATCCGCTTGGTCTGCCCTG	ccaagcggatGCGGATCCACAAGGGAAAC	tcatccaagtctgaggctttcgaaGGCTTGACAGCACCTGCAG	BstBI
11	757C>T	R253W	0.133	0.010	No	tacaaggatgacgataagttcgaaCTCGAGCCCGGGGAATTC	ttgtggatccACGTCCGCTTGGTCTGCC	aagcggacgtGGATCCACAAGGGAAACAGC	tcatccaagtctgaggctttcgaaGGCTTGACAGCACCTGCAG	BstBI
12	797T>G	L266R	0.152	0.012	No	tacaaggatgacgataagttcgaaCTCGAGCCCGGGGAATTC	gttgaagaaaCGAGTCTCATTGAAGAGTGGG	atgagactcgTTTCTTCAACTTGTTTGACTCTCCTG	tcatccaagtctgaggctttcgaaGGCTTGACAGCACCTGCAG	BstBI
13	857T>A	V286E	0.171	0.014	No	tacaaggatgacgataagttcgaaCTCGAGCCCGGGGAATTC	agagtctaccTCCGTGATAAAGATGGGCTC	ttatcacggaGGTAGACTCTCGTTCTCTCAGG	tcatccaagtctgaggctttcgaaGGCTTGACAGCACCTGCAG	BstBI
14	865T>C	S289P	0.114	0.015	No	tacaaggatgacgataagttcgaaCTCGAGCCCGGGGAATTC	agagaacgagGGTCTACCACCGTGATAAAGATG	gtggtagaccCTCGTTCTCTCAGGACAGATGC	tcatccaagtctgaggctttcgaaGGCTTGACAGCACCTGCAG	BstBI
15	895G>A	G299R	0.160	0.007	No	tacaaggatgacgataagttcgaaCTCGAGCCCGGGGAATTC	cggaactcccTGAGGAGAGCATCTGTCCTG	gctctcctcaGGGAGTTCCGGATGGACG	tcatccaagtctgaggctttcgaaGGCTTGACAGCACCTGCAG	BstBI
16	895G>T	G299W	0.098	0.014	No	tacaaggatgacgataagttcgaaCTCGAGCCCGGGGAATTC	cggaactcccAGAGGAGAGCATCTGTCCTG	gctctcctctGGGAGTTCCGGATGGACG	tcatccaagtctgaggctttcgaaGGCTTGACAGCACCTGCAG	BstBI
17	896G>A	G299E	0.165	0.009	No	tacaaggatgacgataagttcgaaCTCGAGCCCGGGGAATTC	ccggaactccTCGAGGAGAGCATCTGTCC	ctctcctcgaGGAGTTCCGGATGGACGTG	tcatccaagtctgaggctttcgaaGGCTTGACAGCACCTGCAG	BstBI
18	965T>C	L322P	0.189	0.018	No	tacaaggatgacgataagttcgaaCTCGAGCCCGGGGAATTC	gtctgagagcGGCAGCCACTTCCTGAGATAG	agtggctgccGCTCTCAGACCCTGATGAC	tcatccaagtctgaggctttcgaaGGCTTGACAGCACCTGCAG	BstBI
19	1020C>A	S340R	0.157	0.041	No	tacaaggatgacgataagttcgaaCTCGAGCCCGGGGAATTC	gcacacaaagTCTTGTTTTCAGGTAGCCTCTG	gaaaacaagaCTTTGTGTGCTGGGGCCTG	tcatccaagtctgaggctttcgaaGGCTTGACAGCACCTGCAG	BstBI
20	1031T>C	L344P	0.143	0.010	No	tacaaggatgacgataagttcgaaCTCGAGCCCGGGGAATTC	cccaggccccGGCACACAAAGGCTTGTTTTC	tttgtgtgccGGGGCCTGGGGACGAAGC	tcatccaagtctgaggctttcgaaGGCTTGACAGCACCTGCAG	BstBI
21	1120G>C	V374L	0.541	0.011	Yes	tacaaggatgacgataagttcgaaCTCGAGCCCGGGGAATTC	cgcagggctaGGCCTGTGGGCCGGAGCA	cccacaggccTAGCCCTGCGAGGAGCCC	tcatccaagtctgaggctttcgaaGGCTTGACAGCACCTGCAG	BstBI
22	1168G>T	D390Y	0.062	0.009	No	tacaaggatgacgataagttcgaaCTCGAGCCCGGGGAATTC	tgcggcaagtACTCGGCCCGGAAGACCT	cgggccgagtACTTGCCGCAGATGGACG	tcatccaagtctgaggctttcgaaGGCTTGACAGCACCTGCAG	BstBI
23	1277G>T	G426V	0.076	0.008	No	tacaaggatgacgataagttcgaaCTCGAGCCCGGGGAATTC	cagcattttcACCGCAAAGCTGACCTCC	gctttgcggtGAAAATGCTGTGCAGCAAGATC	tcatccaagtctgaggctttcgaaGGCTTGACAGCACCTGCAG	BstBI
24	1343T>C	L448P	0.086	0.013	No	tacaaggatgacgataagttcgaaCTCGAGCCCGGGGAATTC	catggcaggcGGTGTGATGTTCTGGTTCCAC	acatcacaccGCCTGCCATGTTTCCCTC	tcatccaagtctgaggctttcgaaGGCTTGACAGCACCTGCAG	BstBI
25	1351A>G	M451V	0.815	0.062	Yes	tacaaggatgacgataagttcgaaCTCGAGCCCGGGGAATTC	gagggaaacaCGGCAGGCAGTGTGATGTTC	ctgcctgccgTGTTTCCCTCCATGTGCG	tcatccaagtctgaggctttcgaaGGCTTGACAGCACCTGCAG	BstBI
26	1369G>A	E457K	0.794	0.060	Yes	tacaaggatgacgataagttcgaaCTCGAGCCCGGGGAATTC	ctcattttttTGCACATGGAGGGAAACATG	tccatgtgcaAAAAAATGAGGATTCGTATCATAGACTGGG	tcatccaagtctgaggctttcgaaGGCTTGACAGCACCTGCAG	BstBI
27	1402C>T	R468C	0.783	0.069	Yes	tacaaggatgacgataagttcgaaCTCGAGCCCGGGGAATTC	tgagtcaggcAGTCCCAGTCTATGATACGAATCC	gactgggactGCCTGACTCACAATGACATCG	tcatccaagtctgaggctttcgaaGGCTTGACAGCACCTGCAG	BstBI
28	1555G>C	G519R	0.086	0.015	No	gtgcgaaaaaatgaggattcgtatcATAGACTGGGACCGCCTG	tagcagggccGAAAAGTGGGGAGGAAGC	cccacttttcGGCCCTGCTACATCAACC	cgatggcggtacgctggagcgctGGCTTGATGTTCTGAGGAAC	AfeI/BsaBI
29	1663C>T	R555W	0.088	0.012	No	gtgcgaaaaaatgaggattcgtatcATAGACTGGGACCGCCTG	agcagaagccAGCCACGATAAGCCACAC	tatcgtggctGGCTTCTGCTCTCCCTGG	cgatggcggtacgctggagcgctGGCTTGATGTTCTGAGGAAC	AfeI/BsaBI
30	1667T>C	L556P	0.087	0.012	No	gtgcgaaaaaatgaggattcgtatcATAGACTGGGACCGCCTG	ggagagcagaGGCCGGCCACGATAAGCC	gtggccggccTCTGCTCTCCCTGGAGAC	cgatggcggtacgctggagcgctGGCTTGATGTTCTGAGGAAC	AfeI/BsaBI
31	1852G>A	G618R	0.094	0.017	No	gtgcgaaaaaatgaggattcgtatcATAGACTGGGACCGCCTG	ccgtagttccTGATGCTGACCTCAAACTG	gtcagcatcaGGAACTACGGGAACAAGTTC	cgatggcggtacgctggagcgctGGCTTGATGTTCTGAGGAAC	AfeI/BsaBI
32	1852G>T	G618W	0.092	0.016	No	gtgcgaaaaaatgaggattcgtatcATAGACTGGGACCGCCTG	ccgtagttccAGATGCTGACCTCAAACTG	gtcagcatctGGAACTACGGGAACAAGTTC	cgatggcggtacgctggagcgctGGCTTGATGTTCTGAGGAAC	AfeI/BsaBI
33	1861G>C	G621R	0.072	0.008	No	gtgcgaaaaaatgaggattcgtatcATAGACTGGGACCGCCTG	aacttgttccGGTAGTTCCCGATGCTGAC	gggaactaccGGAACAAGTTCGACATGAC	cgatggcggtacgctggagcgctGGCTTGATGTTCTGAGGAAC	AfeI/BsaBI
34	1877T>C	M626T	0.871	0.111	Yes	gtgcgaaaaaatgaggattcgtatcATAGACTGGGACCGCCTG	caggcaggtcGTGTCGAACTTGTTCCCG	agttcgacacGACCTGCCTGCCGCTGGC	cgatggcggtacgctggagcgctGGCTTGATGTTCTGAGGAAC	AfeI/BsaBI
35	2105C>T	T702M	0.240	0.028	Yes	gtgcgaaaaaatgaggattcgtatcATAGACTGGGACCGCCTG	cacgtcctccatggagcactgcgccttc	agtgctccatggaggacgtggactcgctg	cgatggcggtacgctggagcgctGGCTTGATGTTCTGAGGAAC	AfeI/BsaBI
36	2162G>C	S721T	0.267	0.052	Yes	gtgcgaaaaaatgaggattcgtatcATAGACTGGGACCGCCTG	cagaggctgggtgcagcctgcgatgagc	caggctgcacccagcctctgggtgacatc	cgatggcggtacgctggagcgctGGCTTGATGTTCTGAGGAAC	AfeI/BsaBI
37	2192C>G	P731R	0.869	0.057	Yes	gtgcgaaaaaatgaggattcgtatcATAGACTGGGACCGCCTG	ggtggcagagCGTGTCTCATGGATGTCAC	atgagacacgCTCTGCCACCCACCTGGA	cgatggcggtacgctggagcgctGGCTTGATGTTCTGAGGAAC	AfeI/BsaBI
38	2367C>A	S789R	0.088	0.011	No	gtgcgaaaaaatgaggattcgtatcATAGACTGGGACCGCCTG	tgtccggcagTCTGTTCTGGGGCTCCTC	ccagaacagaCTGCCGGACATCGTCATC	cgatggcggtacgctggagcgctGGCTTGATGTTCTGAGGAAC	AfeI/BsaBI
39	2408G>A	R803H	0.484	0.086	Yes	gtgcgaaaaaatgaggattcgtatcATAGACTGGGACCGCCTG	gtatgccacaTGCTTGTCTCCCTGCAGC	gagacaagcaTGTGGCATACCAGCGGGTG	cgatggcggtacgctggagcgctGGCTTGATGTTCTGAGGAAC	AfeI/BsaBI
40	2500A>G	I834V	0.910	0.084	Yes	gtgcgaaaaaatgaggattcgtatcATAGACTGGGACCGCCTG	ttcagaaagaCTGTCTGTAGCTTCCCAC	ctacagacagTCTTTCTGAAATATCCGATGG	cgatggcggtacgctggagcgctGGCTTGATGTTCTGAGGAAC	AfeI/BsaBI
41	2516C>T	P839L	0.589	0.023	Yes	gtgcgaaaaaatgaggattcgtatcATAGACTGGGACCGCCTG	cttctccatcAGATATTTCAGAAAGATTGTCTGTAG	tgaaatatctGATGGAGAAGGTGCCTGG	cgatggcggtacgctggagcgctGGCTTGATGTTCTGAGGAAC	AfeI/BsaBI
42	2638G>A	E880K	0.125	0.010	No	gtgcgaaaaaatgaggattcgtatcATAGACTGGGACCGCCTG	tcataggtttTAGCAAAGACAGACAGCTTC	gtctttgctaAAACCTATGAGAACGAGACTAAG	cgatggcggtacgctggagcgctGGCTTGATGTTCTGAGGAAC	AfeI/BsaBI
43	2641A>C	T881P	0.269	0.053	No	gtgcgaaaaaatgaggattcgtatcATAGACTGGGACCGCCTG	ttctcataggGTTCAGCAAAGACAGACAG	tttgctgaacCCTATGAGAACGAGACTAAGTTG	cgatggcggtacgctggagcgctGGCTTGATGTTCTGAGGAAC	AfeI/BsaBI
44	2690C>T	T897M	0.270	0.044	Yes	gtgcgaaaaaatgaggattcgtatcATAGACTGGGACCGCCTG	ggtgaggcccATTGTGCCCCAGTTCCCAAC	ggggcacaatGGGCCTCACCTACCCCAA	cgatggcggtacgctggagcgctGGCTTGATGTTCTGAGGAAC	AfeI/BsaBI
45	2776T>A	W926R	0.189	0.028	No	gtgcgaaaaaatgaggattcgtatcATAGACTGGGACCGCCTG	tctccagcccTGGTCCAGCCGGCCGAGG	ggctggaccaGGGCTGGAGATTGGTTCG	cgatggcggtacgctggagcgctGGCTTGATGTTCTGAGGAAC	AfeI/BsaBI
46	2790G>C	W930C	0.080	0.007	No	gtgcgaaaaaatgaggattcgtatcATAGACTGGGACCGCCTG	gacacacgaaGCAATCTCCAGCCCAGGTC	tggagattgcTTCGTGTGTCCGGAGAAG	cgatggcggtacgctggagcgctGGCTTGATGTTCTGAGGAAC	AfeI/BsaBI
47	2810C >T	T937I	0.688	0.115	Yes	gtgcgaaaaaatgaggattcgtatcATAGACTGGGACCGCCTG	atggagcagaATCTTCTCCGGACACACG	cggagaagatTCTGCTCCATGACATGGAC	cgatggcggtacgctggagcgctGGCTTGATGTTCTGAGGAAC	AfeI/BsaBI
48	2875C>T	R959W	0.126	0.008	No	gtgcgaaaaaatgaggattcgtatcATAGACTGGGACCGCCTG	ccgggaagccAGGTCTGGTTCTCAAACACC	aaccagacctGGCTTCCCGGAGGCCAGT	cgatggcggtacgctggagcgctGGCTTGATGTTCTGAGGAAC	AfeI/BsaBI
49	2902A>T	M968L	0.657	0.051	Yes	gtgcgaaaaaatgaggattcgtatcATAGACTGGGACCGCCTG	ttgtcactcaAGTAGATCCACTGGCCTC	tggatctactTGAGTGACAACTACACCG	cgatggcggtacgctggagcgctGGCTTGATGTTCTGAGGAAC	AfeI/BsaBI
50	2929G>A	G977R	0.834	0.100	Yes	gtgcgaaaaaatgaggattcgtatcATAGACTGGGACCGCCTG	accttctcccTGTTCACATCGGTGTAGTTG	gatgtgaacaGGGAGAAGGTGCTTCCCAAG	cgatggcggtacgctggagcgctGGCTTGATGTTCTGAGGAAC	AfeI/BsaBI
51	2974T>C	W992R	0.168	0.039	No	gtgcgaaaaaatgaggattcgtatcATAGACTGGGACCGCCTG	tcccacttccGGCCCAGTGGGCACTCAA	ccactgggctGGAAGTGGGAAGATGAGG	cgatggcggtacgctggagcgctGGCTTGATGTTCTGAGGAAC	AfeI/BsaBI
52	2980T>A	W994R	0.030	0.023	No	gtgcgaaaaaatgaggattcgtatcATAGACTGGGACCGCCTG	tcatcttcccTCTTCCAGCCCAGTGGGC	ggctggaagaGGGAAGATGAGGAATGGTC	cgatggcggtacgctggagcgctGGCTTGATGTTCTGAGGAAC	AfeI/BsaBI
53	2997G>T	W999C	0.080	0.007	No	gtgcgaaaaaatgaggattcgtatcATAGACTGGGACCGCCTG	ggtctgtggaACATTCCTCATCTTCCCACTTC	tgaggaatgtTCCACAGACCTCAACCGG	cgatggcggtacgctggagcgctGGCTTGATGTTCTGAGGAAC	AfeI/BsaBI

Table S1. List of DYSF^{PMMs} used in this study, related to Figure 1, Figure 2, and Figure S3. Included are results of 2A-assays in HEK cells, ICC results of DYSF localization in HEK cells, and oligonucleotides used for the construction of DYSF^{PMMs} expression vectors.

Table S1:DYSF^{PMMs} Engineering and HEK Cell Results (continued)										
PMM			Assay Result			Oligonucleotides used for generating LV-TRE-DYSFPMMs-T2A-DsRed				
			2A		ICC	PCR1		PCR2		
Clone #	Missense Mutation	Protein Change	Mean	S.D.	DYSF PM Localized?	F1	R1	F2	R2	Vector Digestion Enzyme(s)
54	3031G>C	G1011R	0.088	0.011	No	ctgctccatgacatggacgcCGGTCACCTGAGCTTCGTG	tactcccagcGTTGCTCATCGACAGCCC	gatgagcaacGCTGGGAGTATAGCATCAC	cctgttctccaggtcgacgaCCGTCTCACCGATCTTTTC	PshI
55	3041A>G	Y1014C	0.104	0.028	No	ctgctccatgacatggacgcCGGTCACCTGAGCTTCGTG	ggtgatgctaCACTCCCAGCCTTGCTCATC	gctgggagtgTAGCATCACCATCCCCCC	cctgttctccaggtcgacgaCCGTCTCACCGATCTTTTC	PshI
56	3064C>T	R1022W	0.748	0.039	Yes	ctgctccatgacatggacgcCGGTCACCTGAGCTTCGTG	ttcggcttccACTCCGGGGGGATGGTGA	cccccggagtGGAAGCCGAAGCACTGGG	cctgttctccaggtcgacgaCCGTCTCACCGATCTTTTC	PshI
57	3065G>A	R1022Q	0.956	0.094	Yes	ctgctccatgacatggacgcCGGTCACCTGAGCTTCGTG	cttcggcttcTGCTCCGGGGGGATGGTG	ccccggagcaGAAGCCGAAGCACTGGGTC	cctgttctccaggtcgacgaCCGTCTCACCGATCTTTTC	PshI
58	3113G>C	R1038P	0.087	0.013	No	ctgctccatgacatggacgcCGGTCACCTGAGCTTCGTG	gcgccgccgtGGGTGTGTGTAGTACATCTTCTC	acacacacccACGGCGGCGCTGGGTGCG	cctgttctccaggtcgacgaCCGTCTCACCGATCTTTTC	PshI
59	3113G>A	R1038Q	0.107	0.006	No	ctgctccatgacatggacgcCGGTCACCTGAGCTTCGTG	gcgccgccgtTGGTGTGTGTAGTACATCTTCTCA	acacacaccaACGGCGGCGCTGGGTGCG	cctgttctccaggtcgacgaCCGTCTCACCGATCTTTTC	PshI
60	3116G>T	R1039L	0.093	0.011	No	ctgctccatgacatggacgcCGGTCACCTGAGCTTCGTG	ccagcgccgcAGTCGGTGTGTGTAGTACATCTTC	cacaccgactGCGGCGCTGGGTGCGCCT	cctgttctccaggtcgacgaCCGTCTCACCGATCTTTTC	PshI
61	3118C>T	R1040W	0.067	0.015	No	ctgctccatgacatggacgcCGGTCACCTGAGCTTCGTG	acccagcgccACCGTCGGTGTGTGTAGTAC	caccgacggtGGCGCTGGGTGCGCCTGC	cctgttctccaggtcgacgaCCGTCTCACCGATCTTTTC	PshI
62	3121C>T	R1041C	0.875	0.038	Yes	ctgctccatgacatggacgcCGGTCACCTGAGCTTCGTG	cgcacccagcACCGCCGTCGGTGTGTGT	cgacggcggtGCTGGGTGCGCCTGCGCA	cctgttctccaggtcgacgaCCGTCTCACCGATCTTTTC	PshI
63	3137G>A	R1046H	0.086	0.011	No	ctgctccatgacatggacgcCGGTCACCTGAGCTTCGTG	atccctcctgTGCAGGCGCACCCAGCGC	tgcgcctgcaCAGGAGGGATCTCAGCCAAATG	cctgttctccaggtcgacgaCCGTCTCACCGATCTTTTC	PshI
64	3355G>A	V1119M	0.848	0.050	Yes	ctgctccatgacatggacgcCGGTCACCTGAGCTTCGTG	tcatccatcaTGCCGCCCAGGGCCCCCT	ctgggcggcaTGATGGATGACAAGAGTG	cctgttctccaggtcgacgaCCGTCTCACCGATCTTTTC	PshI
65	3445G>A	G1149R	0.271	0.061	Yes	ctgctccatgacatggacgcCGGTCACCTGAGCTTCGTG	tagcggttccTATAGTCGAATATGCAGGAAATCGT	ttcgactataGGAACCGCTACCATCTAC	cctgttctccaggtcgacgaCCGTCTCACCGATCTTTTC	PshI
66	3466T>C	C1156R	0.160	0.031	No	ctgctccatgacatggacgcCGGTCACCTGAGCTTCGTG	tacatgtagcGGCGTAGATGGTAGCGGTTC	catctacgccGCTACATGTACCAGGCCC	cctgttctccaggtcgacgaCCGTCTCACCGATCTTTTC	PshI
67	3487G>A	D1163N	0.806	0.038	Yes	ctgctccatgacatggacgcCGGTCACCTGAGCTTCGTG	gcagccaggtTCCGGGCCTGGTACATGTAG	caggcccggaACCTGGCTGCGATGGACAAG	cctgttctccaggtcgacgaCCGTCTCACCGATCTTTTC	PshI
68	3624C>G	I1208M	0.859	0.055	Yes	ctgctccatgacatggacgcCGGTCACCTGAGCTTCGTG	caaagatctcCATCTCGTAGAAGATGAGCGTC	ctacgagatgGAGATCTTTGGCGAGCCG	cctgttctccaggtcgacgaCCGTCTCACCGATCTTTTC	PshI
69	3683T>C	L1228P	0.028	0.020	No	ctgctccatgacatggacgcCGGTCACCTGAGCTTCGTG	atggtcgtacGGCTCCACCACAATGCTG	tggtggagccGTACGACCATGACACTTATG	cctgttctccaggtcgacgaCCGTCTCACCGATCTTTTC	PshI
70	3721G>C	G1241R	0.061	0.026	No	ctgctccatgacatggacgcCGGTCACCTGAGCTTCGTG	atgcagcgacGCATAAACTCGTCTGCACC	gagtttatgcGTCGCTGCATCTGTCAAC	cctgttctccaggtcgacgaCCGTCTCACCGATCTTTTC	PshI
71	3851T>C	I1284T	0.873	0.066	Yes	ctgctccatgacatggacgcCGGTCACCTGAGCTTCGTG	aatatggtggGTGGCCGGCTTCTCTCTC	agccggccacCCACCATATTCCTGGTTTTG	cctgttctccaggtcgacgaCCGTCTCACCGATCTTTTC	PshI
72	3892A>G	I1298V	0.668	0.031	Yes	ctgctccatgacatggacgcCGGTCACCTGAGCTTCGTG	tcatccaggaCCCTTGATGTCTCCTGCAC	acatcaagggTCCTGGATGAGTCTGAGG	cctgttctccaggtcgacgaCCGTCTCACCGATCTTTTC	PshI
73	3967C>G	Q1323E	0.850	0.074	Yes	ctgctccatgacatggacgcCGGTCACCTGAGCTTCGTG	ttgatgttctCAGGAACCATGTAGATGTTGGC	atggttcctgAGAACATCAAGCCAGCGC	cctgttctccaggtcgacgaCCGTCTCACCGATCTTTTC	PshI
74	3992G>T	R1331L	0.582	0.073	Yes	ctgctccatgacatggacgcCGGTCACCTGAGCTTCGTG	gatggcggtaAGCTGGAGCGCTGGCTTG	cgctccagctTACCGCCATCGAGATCCTG	cctgttctccaggtcgacgaCCGTCTCACCGATCTTTTC	PshI
75	4004A>G	E1335G	0.229	0.017	No	ctgctccatgacatggacgcCGGTCACCTGAGCTTCGTG	tgccaggatcCCGATGGCGGTACGCTGG	ccgccatcggGATCCTGGCATGGGGCCT	cctgttctccaggtcgacgaCCGTCTCACCGATCTTTTC	PshI
76	4003G>A	E1335K	0.130	0.008	No	ctgctccatgacatggacgcCGGTCACCTGAGCTTCGTG	gccaggatctTGATGGCGGTACGCTGGAG	accgccatcaAGATCCTGGCATGGGGCC	cctgttctccaggtcgacgaCCGTCTCACCGATCTTTTC	PshI
77	4022T>C	L1341P	0.084	0.013	No	ctgctccatgacatggacgcCGGTCACCTGAGCTTCGTG	catgttccgcGGGCCCCATGCCAGGATC	catggggcccGCGGAACATGAAGAGTTACC	cctgttctccaggtcgacgaCCGTCTCACCGATCTTTTC	PshI
78	4024C>T	R1342W	0.495	0.049	Yes	ctgctccatgacatggacgcCGGTCACCTGAGCTTCGTG	ttcatgttccACAGGCCCCATGCCAGGA	tggggcctgtGGAACATGAAGAGTTACCAG	cctgttctccaggtcgacgaCCGTCTCACCGATCTTTTC	PshI
79	4052A>G	N1351S	0.442	0.095	Yes	ctgctccatgacatggacgcCGGTCACCTGAGCTTCGTG	ggaggagatgCTGGCCAGCTGGTAACTC	agctggccagCATCTCCTCCCCCAGCCTC	cctgttctccaggtcgacgaCCGTCTCACCGATCTTTTC	PshI
80	4199C>G	P1400R	0.401	0.081	Yes	ctgctccatgacatggacgcCGGTCACCTGAGCTTCGTG	gacggtgatgCGGGGGCAGTAGAGCTCC	actgcccccgCATCACCGTCAAGGTCATC	cctgttctccaggtcgacgaCCGTCTCACCGATCTTTTC	PshI
81	4253G>A	G1418D	0.156	0.036	No	ctgctccatgacatggacgcCGGTCACCTGAGCTTCGTG	ggtacactggTCCACCACAGGCCGGCGG	ctgtggtggaCCAGTGTACCATCCGCTC	cctgttctccaggtcgacgaCCGTCTCACCGATCTTTTC	PshI
82	4354C>A	P1452T	0.791	0.071	Yes	ctgctccatgacatggacgcCGGTCACCTGAGCTTCGTG	ctgtggtggaCCAGTGTACCATCCGCTC	ctactcagtaCTGGGGAAGACGTGCTCATC	cctgttctccaggtcgacgaCCGTCTCACCGATCTTTTC	PshI
83	4450T>C	S1484P	0.121	0.009	No	ctgctccatgacatggacgcCGGTCACCTGAGCTTCGTG	tcccctatggGGGCAAAGAATTTGCTCCAC	ttctttgcccCCATAGGGGAGAGGGAAAAG	cctgttctccaggtcgacgaCCGTCTCACCGATCTTTTC	PshI
84	4577A>C	K1526T	0.715	0.067	Yes	ctgctccatgacatggacgcCGGTCACCTGAGCTTCGTG	ccggtacagcGTGAAGGTGTTACAAAAGTCAGAC	acaccttcacGCTGTACCGGGGCAAGAC	cctgttctccaggtcgacgaCCGTCTCACCGATCTTTTC	PshI
85	4742G>A	R1581H	0.756	0.077	Yes	ctgctccatgacatggacgcCGGTCACCTGAGCTTCGTG	aatgtagataTGGACCAAGCACTCCTGG	gcttggtccaTATCTACATTGTCCGAGCATTTG	cctgttctccaggtcgacgaCCGTCTCACCGATCTTTTC	PshI
86	4794G>T	K1598N	0.753	0.085	Yes	ctgctccatgacatggacgcCGGTCACCTGAGCTTCGTG	aaggatcacaATTTCCATTGGGGTCCTTGG	caatggaaatTGTGATCCTTACATCAAGATC	cctgttctccaggtcgacgaCCGTCTCACCGATCTTTTC	PshI
87	4820T>C	I1607T	0.825	0.079	Yes	cctcagaacatcaagccagcGCTCCAGCGTACCGCCAT	tttcttccctGTGGAGATCTTGATGTAAGGATCACA	agatctccacAGGGAAGAAATCAGTGAG	tatcgatgcggccgcgctagcTTACAATTCGTCGTGCTTG	BmtI/AfeI
88	4882G>A	G1628R	0.048	0.024	No	cctcagaacatcaagccagcGCTCCAGCGTACCGCCAT	aacatctttcTAAATACGGGCTCCAGCGTG	cccgtatttaGAAAGATGTTCGAGCTGAC	tatcgatgcggccgcgctagcTTACAATTCGTCGTGCTTG	BmtI/AfeI
89	4883G>T	G1628V	0.154	0.016	No	cctcagaacatcaagccagcGCTCCAGCGTACCGCCAT	gaacatctttACAAATACGGGCTCCAGCG	ccgtatttgtAAAGATGTTCGAGCTGAC	tatcgatgcggccgcgctagcTTACAATTCGTCGTGCTTG	BmtI/AfeI
90	4934T>A	I1645N	0.100	0.024	No	cctcagaacatcaagccagcGCTCCAGCGTACCGCCAT	atagagagtgTTCTTTAGGTCCTTCTCCAGAGGC	acctaaagaaCACTCTCTATGACTATGAC	tatcgatgcggccgcgctagcTTACAATTCGTCGTGCTTG	BmtI/AfeI
91	5032T>C	C1678R	0.168	0.018	No	cctcagaacatcaagccagcGCTCCAGCGTACCGCCAT	gggagtccacGGCGAGCCCCAAACTTGG	ggggctcgccGTGGACTCCCACAGACCTAC	tatcgatgcggccgcgctagcTTACAATTCGTCGTGCTTG	BmtI/AfeI
92	5077C>T	R1693W	0.176	0.053	No	cctcagaacatcaagccagcGCTCCAGCGTACCGCCAT	agctggtcccACCACTGGTTCGGTCCAG	aaccagtggtGGGACCAGCTCCGCCCCT	tatcgatgcggccgcgctagcTTACAATTCGTCGTGCTTG	BmtI/AfeI
93	5296G>A	E1766K	0.129	0.016	No	cctcagaacatcaagccagcGCTCCAGCGTACCGCCAT	ggccgtgactTCACGTGCTCCGGGACCA	gagcacgtgaAGTCACGGCCCCTCTACAG	tatcgatgcggccgcgctagcTTACAATTCGTCGTGCTTG	BmtI/AfeI
94	5302C>T	R1768W	0.094	0.020	No	cctcagaacatcaagccagcGCTCCAGCGTACCGCCAT	tagaggggccATGACTCCACGTGCTCCG	gtggagtcatGGCCCCTCTACAGCCCCC	tatcgatgcggccgcgctagcTTACAATTCGTCGTGCTTG	BmtI/AfeI
95	5306C>A	P1769H	0.669	0.091	Yes	cctcagaacatcaagccagcGCTCCAGCGTACCGCCAT	gctgtagaggTGCCGTGACTCCACGTGC	agtcacggcaCCTCTACAGCCCCCTGCAG	tatcgatgcggccgcgctagcTTACAATTCGTCGTGCTTG	BmtI/AfeI
96	5309T>C	L1770P	0.107	0.019	No	cctcagaacatcaagccagcGCTCCAGCGTACCGCCAT	caggggctgtagGGGGGCCGTGACTCCACG	cacggcccccCTACAGCCCCCTGCAGCC	tatcgatgcggccgcgctagcTTACAATTCGTCGTGCTTG	BmtI/AfeI
97	5342G>A	G1781E	0.138	0.011	No	cctcagaacatcaagccagcGCTCCAGCGTACCGCCAT	ctgcagcttcTCCTGCTCGATGTCTGGC	tcgagcaggaGAAGCTGCAGATGTGGGTC	tatcgatgcggccgcgctagcTTACAATTCGTCGTGCTTG	BmtI/AfeI
98	5429G>A	R1810K	0.670	0.090	Yes	cctcagaacatcaagccagcGCTCCAGCGTACCGCCAT	caggaaaaacTTTCTGGCTCTCCGTGGG	gagccagaaaGTTTTTCCTGCGTTGTATTATC	tatcgatgcggccgcgctagcTTACAATTCGTCGTGCTTG	BmtI/AfeI
99	5441G>A	R1814H	0.456	0.032	Yes	cctcagaacatcaagccagcGCTCCAGCGTACCGCCAT	gataatacaaTGCAGGAAAAACCTTCTGGC	ttttcctgcaTTGTATTATCTGGAATACCAG	tatcgatgcggccgcgctagcTTACAATTCGTCGTGCTTG	BmtI/AfeI
100	5444G>T	C1815F	0.023	0.008	No	cctcagaacatcaagccagcGCTCCAGCGTACCGCCAT	ccagataataAAACGCAGGAAAAACCTTCTGG	tcctgcgtttTATTATCTGGAATACCAGAGATG	tatcgatgcggccgcgctagcTTACAATTCGTCGTGCTTG	BmtI/AfeI
101	5503A>G	M1835V	0.592	0.063	Yes	cctcagaacatcaagccagcGCTCCAGCGTACCGCCAT	atgtcgctcaCCTTCTCCCCCGTGAGGC	ggggagaaggTGAGCGACATTTATGTGAAAG	tatcgatgcggccgcgctagcTTACAATTCGTCGTGCTTG	BmtI/AfeI
102	5509G>A	D1837N	0.205	0.022	No	cctcagaacatcaagccagcGCTCCAGCGTACCGCCAT	acataaatgtTGCTCATCTTCTCCCCCG	aagatgagcaACATTTATGTGAAAGGTTGG	tatcgatgcggccgcgctagcTTACAATTCGTCGTGCTTG	BmtI/AfeI
103	5518G>A	V1840M	0.161	0.020	No	cctcagaacatcaagccagcGCTCCAGCGTACCGCCAT	caacctttcaTATAAATGTCGCTCATCTTCTCCCC	gacatttataTGAAAGGTTGGATGATTG	tatcgatgcggccgcgctagcTTACAATTCGTCGTGCTTG	BmtI/AfeI
104	5601C>G	F1867L	0.067	0.012	No	cctcagaacatcaagccagcGCTCCAGCGTACCGCCAT	acctccagttCAAGTTGCCTTCACCTCCC	aggcaacttgAACTGGAGGTTCATTTTC	tatcgatgcggccgcgctagcTTACAATTCGTCGTGCTTG	BmtI/AfeI
105	5729T>A	I1910N	0.028	0.010	No	cctcagaacatcaagccagcGCTCCAGCGTACCGCCAT	attgtcccagTTCTGGAACACCACTCGTGC	tgttccagaaCTGGGACAATGACAAGTTC	tatcgatgcggccgcgctagcTTACAATTCGTCGTGCTTG	BmtI/AfeI
106	5902T>C	W1968R	0.032	0.006	No	cctcagaacatcaagccagcGCTCCAGCGTACCGCCAT	cagggccaccGGCCCTTCACTGTTTTCTGC	gtgaagggccGGTGGCCCTGTGTAGCAG	tatcgatgcggccgcgctagcTTACAATTCGTCGTGCTTG	BmtI/AfeI
107	5907G>C	W1969C	0.030	0.010	No	cctcagaacatcaagccagcGCTCCAGCGTACCGCCAT	ctacacagggGCACCAGCCCTTCACTGTTTTC	gggctggtgcCCCTGTGTAGCAGAAGAG	tatcgatgcggccgcgctagcTTACAATTCGTCGTGCTTG	BmtI/AfeI
108	5908C>T	P1970S	0.183	0.055	No	cctcagaacatcaagccagcGCTCCAGCGTACCGCCAT	gctacacaggACCACCAGCCCTTCACTG	ggctggtggtCCTGTGTAGCAGAAGAGG	tatcgatgcggccgcgctagcTTACAATTCGTCGTGCTTG	BmtI/AfeI
109	6008G>A	G2003D	0.513	0.032	Yes	cctcagaacatcaagccagcGCTCCAGCGTACCGCCAT	ccggccctggTCAGCAGGCCGCTCCTCA	ggcctgctgaCCAGGGCCGGGATGAGCC	tatcgatgcggccgcgctagcTTACAATTCGTCGTGCTTG	BmtI/AfeI
110	6056G>A	R2019K	0.898	0.066	Yes	cctcagaacatcaagccagcGCTCCAGCGTACCGCCAT	gtcggggcgcTTTGGGTCCTCAAGCTTAGGG	aggacccaaaGCGCCCCGACACCTCCTT	tatcgatgcggccgcgctagcTTACAATTCGTCGTGCTTG	BmtI/AfeI
111	6124C>T	R2042C	0.340	0.052	No	cctcagaacatcaagccagcGCTCCAGCGTACCGCCAT	caccggaaacACCGCCACAGGATGAACTTC	ctgtggcggtGTTTCCGGTGGGCCATCATC	tatcgatgcggccgcgctagcTTACAATTCGTCGTGCTTG	BmtI/AfeI
112	6196G>A	A2066T	0.606	0.067	Yes	cctcagaacatcaagccagcGCTCCAGCGTACCGCCAT	ttcgggaaggTGTAGATGAAGATGGCCAGGAAC	ttcatctacaCCTTCCCGAACTATGCTG	tatcgatgcggccgcgctagcTTACAATTCGTCGTGCTTG	BmtI/AfeI
113	6217A>G	M2073V	0.824	0.024	Yes	cctcagaacatcaagccagcGCTCCAGCGTACCGCCAT	accagcttcaCGGCAGCATAGTTCGGGAAG	tatgctgccgTGAAGCTGGTGAAGCCCTTC	tatcgatgcggccgcgctagcTTACAATTCGTCGTGCTTG	BmtI/AfeI

Table S1 continued. List of DYSF^{PMMs} used in this study, related to Figure 1, Figure 2, and Figure S3. Included are results of 2A-assays in HEK cells, ICC results of DYSF localization in HEK cells, and oligonucleotides used for the construction of DYSF^{PMMs} expression vectors.

Table S2: DYSF^{PMMs} Present in the Homozygous State in Individuals from the Dyferlin Registry			
**DYSF Missense Mutation (36 Variants)**	**# of patients (90 Total)**	**2A-Expression Value**	**Exon Skipping**
**R2019K**	2	0.90	Yes
**R1041C**	1	0.88	
**G977R**	1	0.83	
**I1607T**	3	0.83	
**P1452T**	1	0.79	
**K1526T**	1	0.72	
**R1810K**	1	0.67	Yes
**P1769H**	1	0.67	
**R1331L**	2	0.58	
**R2042C**	4	0.34	
**T252M**	1	0.25	
**V224G**	1	0.22	
**W926R**	1	0.19	
**R1693W**	3	0.18	
**C1156R**	1	0.16	
**V69G**	6	0.16	
**S340R**	2	0.16	
**G1418D**	6	0.16	
**V67D**	1	0.15	
**W52R**	1	0.14	
**R253W**	2	0.13	
**R959W**	2	0.13	
**R1038Q**	2	0.11	
**Y1014C**	11	0.10	
**I1645N**	1	0.10	
**G299W**	1	0.10	
**R1768W**	2	0.09	
**G618R**	2	0.09	
**R555W**	6	0.09	
**L556P**	1	0.09	
**G519R**	2	0.09	
**R1046H**	10	0.09	
**L1341P**	5	0.08	
**G1628R**	1	0.05	
**W1968R**	1	0.03	
**W1969C**	1	0.03	
			**# of variants**
		**2A < 0.25 (No PM localization)**	26 (72%)
		**Exon Skipping Mutants, 2A > 0.25**	2 (6%)
		**2A > 0.25 (PM localization)**	8 (22%)
		**Total**	**36**

Table S2. Homozygous mutations in the Jain Foundation Dysferlin Registry containing *DYSF* ^{PMMs} selected for this study, related to Figure 2. List of 36 DYSF^{PMMs} that exist among 90 of the 327 patients carrying the 113 DYSF^{PMMs} selected for this study. 87 individuals carry a single homozygous PMM, 3 patients are homozygous for both I1607T and Y1014C; 1 patient is homozygous for both R1331L and R253W; in all four cases one of the variants has 2A-expression values >0.25 and the other <0.25. DYSF^{R2042C} is not seen at the PM by ICC assay.

Table S3: Pathogenicity Evidence for DYSF^{PMMs} > 0.25 in 2A-assay							
	Evidence of Non-Pathogenicity						Evidence of Pathogenicity
DYSF Missense Mutation	Seen in Patients with Two Other Known Pathogenic DYSF Variants	Seen in Patients in Combinatio n with Variants Predicted to be Pathogenic by 2A-assay	Patient Confirmed with Another MD	Patients Have Carrier or Normal Dysferlin Protein Levels	Altered Splicing	ClinVar Calls Variant Benign	
L7P						X	
W46R							
P134L		X					
A170E						X	
V374L	X						
M451V				X			
E457K						X	
R468C				X			
M626T		X					
T702M	X						
S721T					X		
P731R							X
R803H				X			
I834V						X	
P839L	X						
T881P					X		
T897M		X					
T937I							
M968L				X			
R1022Q			X				
R1022W							
V1119M				X			
G1149R	X						
D1163N			X				
I1208M				X			
I1284T				X			
I1298V	X						
Q1323E			X				
R1342W		X					
N1351S				X			
P1400R		X					
R1581H		X					
K1598N					X		
R1814H							X
M1835V					X		
G2003D							X
A2066T							X
M2073V							X
Total	5	6	3	8	4	4	5
%	13%	16%	8%	21%	11%	11%	13%

Table S3. Supportive evidence for pathogenicity calls on DYSF^{PMMs} where 2A-assay and ICC show PM-localized DYSF, related to Figure 2. The majority of listed DYSF^{PMMs} are found as heterozygous alleles and have evidence of non-pathogenicity. R1814H, G2003D, and P731R are found with other known pathogenic DYSF variants in individuals that show disease range dysferlin protein levels. A2066T and M2073V are each found in trans with pathogenic nonsense mutations in individuals in the registry. No additional pathogenicity evidence is available for L7P, W46R, T937I, or R1022W. ClinVar is the NCBI NIH archive of the relationships among human variations and phenotypes, and has evidence-based calls on pathogenicity.
